# Effects of Hazelnut Consumption on Cardiometabolic Risk Factors and Acceptance: A Systematic Review

**DOI:** 10.3390/ijerph19052880

**Published:** 2022-03-01

**Authors:** Rachel Brown, Lara Ware, Siew Ling Tey

**Affiliations:** Department of Human Nutrition, University of Otago, Dunedin 9054, New Zealand; rachel.brown@otago.ac.nz (R.B.); lara.ware@otago.ac.nz (L.W.)

**Keywords:** hazelnuts, blood lipids and lipoproteins, apolipoproteins, body weight and composition, blood pressure, glycaemia, oxidative stress, inflammation, endothelial function, acceptance

## Abstract

Despite being rich sources of monounsaturated fat and a number of vitamins, minerals, and phytonutrients, hazelnuts have received less attention than some other nut types. A qualitative systematic review was carried out to determine the effects of hazelnut consumption on acceptance and markers of cardiometabolic health, including blood lipids and lipoproteins, apolipoproteins A1 and B100, body weight and composition, blood pressure, glycemia, antioxidant status, oxidative stress, inflammation, and endothelial function. In total, 22 intervention studies (25 publications) met our inclusion criteria. The findings indicate some improvements in cardiometabolic risk factors; however, limitations in study design mean interpretation is problematic. The inclusion of hazelnuts in the diet did not adversely affect body weight and composition. Acceptance of hazelnuts remained stable over time confirming nut consumption guidelines are feasible and sustainable. Future studies using more robust study designs in a variety of populations are required to draw more definitive conclusions on the health benefits of hazelnut consumption.

## 1. Introduction

Observations from large cohort studies indicate regular nut consumption is associated with a reduction in the risk of total mortality and a number of chronic diseases, such as cardiovascular disease and certain cancers [1,2,3]. Studies on diabetes, hypertension, and stroke are equivocal, with the majority showing no significant associations [4,5,6,7]. Although nuts are high in energy and fat, observational studies report that nut consumers are leaner than non-nut consumers [8,9]. Additionally, longitudinal studies report nut consumption is associated with a lower risk of overweight and obesity, weight gain, and deposition of abdominal adiposity [10,11].

Randomised controlled trials have shown improvements in risk factors of chronic disease with regular nut consumption. For example, total cholesterol and low-density lipoprotein cholesterol (LDL-C) are consistently lowered by regular nut consumption, with reductions more pronounced in those with elevated cholesterol concentrations [12,13,14]. Findings on blood pressure and biomarkers of oxidation, inflammation, and endothelial function are mixed, with some showing positive effects, while others report no effect [13,15,16,17,18]. In support of observational studies, intervention studies have found that adding nuts to the usual diet results in no weight gain or less than expected weight gain given the additional calories provided by nuts [11,19].

Despite being the second-largest nut produced worldwide, hazelnuts have received less attention regarding their health benefits than some other nut types [20,21]. Hazelnuts are high in monounsaturated fats and are a source of fibre, vitamin E, folate, potassium, copper, manganese, phosphorous, magnesium, and phytosterols [21]. They also contain high amounts of flavonoids and phenolic compounds, especially in their skin [22,23].

While there are many recent comprehensive systematic reviews and meta-analyses on the health effects of almonds [24], cashews [25], pistachios [26,27], and walnuts [28,29,30], only one systematic review and meta-analysis has reviewed the evidence on hazelnuts. This review published in 2016 reported the effects of hazelnut consumption on blood lipids and body weight [31]. Only three of the nine studies included in this review were randomised controlled trials. The meta-analysis of these three studies showed a significant reduction in LDL-C and a tendency for a reduction in total cholesterol, but no significant changes in high-density lipoprotein cholesterol (HDL-C), triglycerides (TAG), or body mass index (BMI).

We aimed to extend this review to include studies that have been published since its publication and to expand the outcomes to also include apolipoproteins, blood pressure, glycaemic response, acceptance, and markers of inflammation, oxidation, and endothelial function.

## 2. Materials and Methods

### 2.1. Search Strategy

The protocol for this systematic review was registered with PROSPERO (registration number CRD42020203171). Medline (via Ovid), PubMed, Scopus, and Google Scholar databases were searched on 29 July 2020. The search was updated on 28 November 2021, but no further studies meeting our eligibility criteria were identified. The search strategy was limited to human studies and articles written in the English language. Reference lists from publications identified by our searches were manually searched to identify relevant research not found in the database searches. Search terms are outlined in Supplementary Material Table S1.

Study selection was then conducted by SLT and RB using Rayyan [32], and any disagreements were resolved by consultation.

### 2.2. Inclusion and Exclusion Criteria

Studies were included if they met the following criteria: were intervention studies in human participants, included hazelnuts, and evaluated at least one of the study outcomes (see Table 1). Studies were excluded if they were non-English language, reviews, expert opinions, theses, animal, or in vitro studies, if the independent effects of hazelnuts could not be assessed, or if hazelnut oil was used as the test food. Our PICOS statement is outlined in Table 1.

### 2.3. Data Extraction

Data extracted included authors, year, study design, participant characteristics, intervention period, treatments (including dose), and outcomes.

### 2.4. Study Quality

This review was undertaken using the principles outlined in the PRISMA 2020 statement [33]. The risk of bias for each study was assessed by all authors using the Cochrane Collaboration Risk of Bias Tool for randomised controlled intervention studies [34] and the Risk of Bias in Non-Randomised Studies—of Interventions (ROBINS-I) for non-randomised intervention studies [35]. 

The Cochrane Collaboration Risk of Bias Tool considers the following domains: selection bias, reporting bias, performance bias, detection bias, attrition bias, and any other identified biases [34]. Each domain was classified as low, high, or unclear risk of bias. Studies with low risk for ≤one domain were classified as poor, studies with a low risk of bias for two domains were classified as fair, and studies with a low risk of bias in at least three domains were classified as good. 

The ROBINS-I tool considers bias in the following domains: confounding, selection of study participants, classification of interventions, deviations from intended interventions, missing data, measurement of outcomes, selection of reported results [35]. Each domain was classified as low, moderate, serious, or critical. Studies for low risk of bias for all domains were classified as low, studies with low or moderate risk of bias for all domains were classified as moderate, studies with serious risk of bias in at least one domain, but not at critical risk of bias in any domain were classified as serious, and studies with critical risk of bias in at least one domain were classified as critical.

## 3. Results

**Figure 1:**
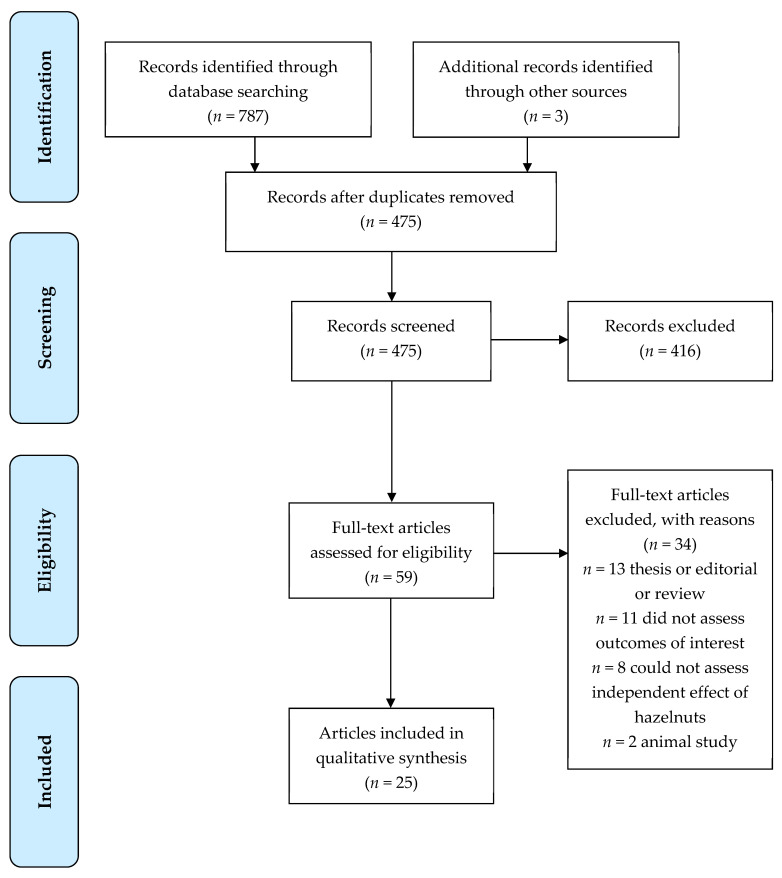
Flow diagram of the literature search process.

The search criteria returned a total of 787 articles. A total of 475 were excluded as duplicates. After abstract review, 58 were included for review. After retrieval of the selected papers, 25 papers (22 studies) were included in the present review (Figure 1). Seven of the studies were conducted in New Zealand, seven in Italy, six in Turkey, one in Iran, and one in the USA.

### 3.1. Risk of Bias

The quality of the methods for the studies is presented in Table 2 and Table 3. Overall, 10 randomised trials were rated as good, one as fair, and one as poor (Table 2). For non-randomised trials, the overall risk of bias for two studies was rated as moderate, and eight were rated as critical (Table 3). 

### 3.2. Blood Lipids and Lipoproteins

In total, 17 studies examined the effects of hazelnut consumption on blood lipids and lipoproteins (Table 4). Different study designs included: randomised parallel (*n* = 6), randomised crossover (*n* = 2), sequential (*n* = 2), double control sandwich (*n* = 2), and single intervention (*n* = 5). Interventions ranged in duration from 2 to 16 weeks. Sample sizes ranged from 15 to 118 and were heterogeneous in nature. For example, nine samples comprised healthy participants, five included those with hyperlipidaemia (including one with children), two included those with type 2 diabetes, and one specifically recruited people with overweight or obesity.

Nine studies compared hazelnut consumption to a no-nut control. Of these, two reported significantly lower total cholesterol and LDL-C [57,58], and three reported significantly higher HDL cholesterol [36,38,57] in the hazelnut group compared to the control. Only one study reported a significant reduction in TAG in the hazelnut group compared to the control [57]. Four studies reported no significant differences in any of the outcomes [39,45,47,50]. 

Two studies included a hazelnut and high carbohydrate treatment. Alphan et al. [51] reported significant decreases in total and LDL-C in the hazelnut group, with significant increases in LDL-C in the high carbohydrate group. However, they failed to report between-group differences. Mercanligil et al. [55] reported significantly higher HDL-C in the hazelnut group compared to the high carbohydrate control.

Of the single intervention studies, three observed reductions in total cholesterol [53,54,60], four in LDL-C [53,54,56,60], while one reported an increase in HDL-C [54], and one an increase in TAG [54].

Two randomised crossover studies compared different forms of hazelnuts. One study compared ground vs. sliced vs. whole nuts [43]. There were no significant differences between treatments, but all three forms were associated with significant reductions in total cholesterol and LDL-C, and significant increases in HDL-C. A further study compared raw vs. roasted hazelnuts [49]. HDL-C was significantly higher following raw hazelnuts, while TAG was significantly lower following the roasted hazelnuts. There were no between-group differences for total cholesterol and LDL-C. Within-group, changes included a significant decrease in LDL-C and a significant increase in HDL-C with raw hazelnut consumption.

Overall, 9 (4 RCTs, 3 single arm, 2 different forms) and 10 (4 RCTs, 4 single arm, 2 different forms) of the 16 studies reported statistically significant reductions in total and LDL-C with hazelnut consumption, respectively. For HDL-C, 7 (4 RCTs, 1 single intervention, 2 different forms) studies reported statistically significant increases. Two (1 RCT, 1 different forms) studies reported significant reductions in TAG while 1 (single intervention) reported a significant increase.

### 3.3. Apolipoproteins A and B

Eight studies examined the effects of hazelnut consumption on apolipoproteins (apo) A and B (Table 5). Study design included randomised parallel (*n* = 1), randomised crossover (*n* = 2), sequential (*n* = 2), double control sandwich (*n* = 1), and single intervention (*n* = 2). Most of the studies were 4 weeks in duration, with one being 2 weeks and one 12 weeks. Sample sizes ranged from 15 to 107 participants. Three studies included healthy participants, three included those with hyperlipidaemia (including mild hyperlipidaemia), one included those with type 2 diabetes, and one specifically recruited people with overweight or obesity.

Two studies compared hazelnut consumption to a no-nut control [47,57]. Tey et al. compared the consumption of two doses of hazelnuts (30 g and 60 g) to a no-nut control in a parallel study [47]. There were no between-group differences. Orem et al. [57] used a double control sandwich model intervention design. Apo A significantly increased after the hazelnut period compared to control I and decreased again after control period II compared to the hazelnut period. Apo B significantly increased after control period II compared to the hazelnut period.

Two studies included a hazelnut and high carbohydrate group using a sequential design [51,55]. Alphan et al. did not report between-group differences, and there were no statistically significant within-group changes [51]. Mercanligil et al. reported no significant differences between the diet groups [55]. 

Of the single intervention studies, Yucesan et al. reported a significant increase in apo A and a significant decrease in apo B [60], while Tey et al. reported no significant changes [59].

Two studies compared different forms of hazelnuts. Tey et al. showed no significant differences in apo A or B between ground, sliced, and whole nuts, but all three forms were associated with significant reductions in apo B [43]. In a further study, they reported no significant differences for raw versus roasted hazelnuts, but both forms significantly increased apo A compared to baseline [49].

Overall, three of the eight studies reported a significant increase in apo A, and four reported a significant reduction in apo B with hazelnut consumption.

### 3.4. Body Weight and Composition

In total, 17 studies examined the effects of hazelnut consumption on body weight and composition (Table 6). Study designs included: randomised parallel (*n* = 5), randomised crossover (*n* = 2), sequential (*n* = 3), double control sandwich (*n* = 2), and single intervention (*n* = 5). Interventions ranged in duration from 4 to 16 weeks. Sample sizes ranged from 15 to 118 and were heterogeneous in nature. For example, nine comprised healthy participants, five included those with hyperlipidaemia (including one with children), two included those with type 2 diabetes and one specifically recruited people with overweight or obesity.

Ten studies compared hazelnut consumption to a no-nut control. Of these, one study, a sequential intervention study, reported a significant increase in hip circumference and lean body mass, and a significant reduction in fat mass, after the hazelnut diet compared to the standard diet [52]. Nine studies found no significant differences between-groups for any outcome, and one failed to report between-group differences [51]. Of the single intervention studies, one reported a reduction in abdominal circumference [53], and one reported an increase in BMI from baseline [56]. Three studies compared different forms of hazelnuts. One study compared hazelnuts with and without skin [39], one study compared ground vs. sliced vs. whole hazelnuts [43], and a third study compared raw vs. roasted hazelnuts [49]. None of these studies reported any change in body composition.

### 3.5. Blood Pressure

In total, seven studies examined the effects of hazelnut consumption on blood pressure (Table 7) [39,47,49,50,53,56,59]. Different study designs included: randomised parallel (*n* = 3), randomised crossover (*n* = 1), and single intervention (*n* = 3). Interventions ranged in duration from 4 to 16 weeks. Sample sizes ranged from 24 to 107 and were heterogeneous in nature. For example, four samples comprised healthy participants, one comprised obese women with hyperlipidaemia, one comprised children and adolescents with hyperlipidaemia, and one specifically recruited people with overweight and obesity.

Three studies compared hazelnut consumption to a no-nut control group [39,47,50], and two compared the consumption of different forms of hazelnuts [39,49]. None of these studies reported significant differences between treatments. Similarly, two single intervention studies reported no significant change in blood pressure following hazelnut consumption [53,56], and one single intervention reported a significant reduction in systolic blood pressure in the total cohort (combining Māori and European participants) [59].

### 3.6. Glycaemia

Nine studies examined glycaemia as an outcome, including one acute study (Table 8). The acute study measured 2 h incremental area under the curve (iAUC) for blood glucose in response to four breads containing no nuts, 30 g of finely sliced nuts, 30 g of defatted hazelnut flour, or 15 g of finely sliced nuts and 15 g of defatted hazelnut flour [40]. The iAUC for blood glucose was significantly lower for all hazelnut-containing breads compared to the no-nut bread.

The longer-term studies used a number of different indices to measure glycaemia. These included glycated haemoglobin (HbA1), fasting blood glucose (FBG), post-prandial blood glucose, fasting insulin, postprandial insulin, and the homeostasis model-insulin resistance (HOMA-IR).

Seven studies examined FBG concentrations. Only one study using a single intervention design showed a significant reduction in FBG [56].

Insulin concentrations were reported in four studies. Orem et al. reported that there was no significant difference in fasting insulin levels between the hazelnut-enriched diet and no nut control diet [57]. In addition, Adamo et al. reported that fasting insulin levels remained stable among those consuming 30 g of peeled hazelnut paste, 30 g of unpeeled hazelnut paste, or 30 g of peeled hazelnuts for breakfast for 2 weeks [36]. Actual changes in insulin were not presented, and no information on insulin levels in other groups receiving a cocoa snack, a combination of cocoa and 30 g peeled hazelnuts, and a no nut control was provided. Two other studies only assessed within-group differences and reported no significant changes in fasting or postprandial insulin concentrations [51,56].

Only one study measured HbA1c, and it should be noted that the intervention was only for 30 days [51]. This study used a sequential design with a high carbohydrate diet and hazelnut diet among 19 people with type 2 diabetes. Between-group differences were not reported, but there was a significant reduction in HbA1c in the hazelnut group.

Two studies assessed insulin resistance using HOMA-IR [36,57]. Adamo et al. did not report specific values, only commenting that HOMA-IR remained stable among those consuming 30 g of peeled hazelnut paste, 30 g of unpeeled hazelnut paste, or 30 g of peeled hazelnuts for breakfast for 2 weeks [36]. Orem reported non-significant differences in HOMA-IR between the hazelnut treatment and no-nut control in their sandwich model study [57].

Overall, the one acute study showed a reduction in iAUC for blood glucose with consumption of hazelnut in a carbohydrate-rich [40]. In studies with a longer intervention, only one of six studies reported lower FBG with hazelnut consumption. Three studies that assessed fasting and/or postprandial insulin showed no significant reductions with hazelnut consumption. One study reported reductions in HbA1c with hazelnut consumption among people with diabetes. Two studies that assessed HOMA-IR reported no significant differences with hazelnut consumption.

### 3.7. Inflammation, Oxidation, and Endothelial Function

Sixteen studies have examined the effects of hazelnut consumption on antioxidant status and/or markers of inflammation, oxidative stress, and/or endothelial function (Table 9).

Nine studies assessed antioxidant status, with two studies reporting upregulation in the expression of genes involved in antioxidant and/or anti-inflammator pathways with hazelnut consumption [52,53]. A further three single intervention studies reported increased antioxidant markers [54,56,60]. Michels et al. reported improvements in some but not all outcomes [56]. Two studies reported no significant differences in alpha-tocopherol after consuming different forms of hazelnuts [43,49], although there was evidence of increases from baseline. A further three studies reported mixed results, with one reporting positive results [57] and two showing no differences between groups [37,47].

One acute study [41] and one chronic study (4 weeks) [52] reported a reduction in oxidised LDL after consuming 40 g of hazelnuts, compared to meals without nuts. A further single intervention reported significant reductions in oxidised LDL compared to baseline [60]. Conversely, there are mixed results when nut interventions are compared to no nut controls. Orem et al. reported significant reductions on oxidised LDL after consuming a hazelnut enriched diet. A further single intervention reported significant decreases in plasma malondialdehyde (MDA) [54]. Conversely, Guaraldi (2018) showed no significant differences in oxidised LDL, DNA strand breaks, and H_2_O_2_ DNA damage, while formamidopyrimidine DNA glycosylase (FPG)-sensitive sites in PBMCs were reduced significantly when hazelnut consumption was compared to a no nut control.

Seven studies looked at the effects of hazelnut consumption on inflammatory markers such as CRP and interleukin-6, with six studies (three RCTs and three single intervention studies) reporting no improvement in inflammatory markers [36,37,47,56,59,60] and one reporting a significant reduction in CRP [57].

One study reported significant increases in peak systolic velocity (PSV) with hazelnut consumption compared to the control group [36]. Mercanligil reported no significant differences in endothelial function measured by doppler ultrasound [55], whereas Orem showed significant improvements [57]. Two RCTs assessed intracellular adhesion molecule-1 (ICAM-1) and vascular adhesion molecule-1 (VCAM-1) [47,57]. Orem showed significant improvements in both markers with 49 to 86 g/d of hazelnuts among people with hypercholesterolaemia [57], whereas Tey et al. showed no significant differences with 30 to 60 g/d of hazelnuts among people with overweight and obesity [47]. 

### 3.8. Sensory Acceptance

Seven studies have measured the effects of repeated consumption of hazelnuts on the desire to consume and overall liking using 100 mm or 150 mm visual analogue scales with exposure ranging from 5 to 84 days (Table 10). Both ratings remained stable over time, except for one dose-response study [47]. This study showed the desire to consume ratings increased over time with 30 g/d of hazelnuts for 12 weeks, whereas the desire to consume and overall liking ratings decreased over time for the 60 g/d groups.

Several studies compared different forms of hazelnuts [40,44,48,49]. Devi et al. incorporated different forms of hazelnuts into bread. Desire to eat and overall liking ratings from highest to lowest were: bread containing 30 g finely sliced hazelnuts, bread containing 15 g finely sliced hazelnuts, and 15 g of defatted hazelnut flour, control bread containing no nuts, and bread containing 30 g defatted hazelnut flour [40].

A further two studies reported desire to consume, and overall liking ratings were highest for whole hazelnuts, followed by sliced hazelnuts, and ground hazelnuts had the lowest ratings [44,48]. 

One study compared acceptance ratings for raw hazelnuts with dry roasted, lightly salted hazelnuts. Both forms of hazelnuts were equally liked [49].

One study compared isocaloric amounts of hazelnuts (42 g/d) with chocolate (50 g/d) and potato crisps (50 g/d). The liking ratings for hazelnuts remained stable over time, whereas the ratings for both chocolate and potato crisps declined significantly [46]. 

## 4. Discussion

Overall, we identified 22 studies (25 papers) that examined the effects of hazelnut consumption on at least one of the outcomes of interest. Many of the studies suffered from methodological flaws, including lack of randomisation, lack of a control group, small samples, short duration, lack of between-group analyses, and poor reporting of findings. These factors may account for some of the inconsistent findings. One finding that was consistent with previous literature on other nut types is the null effect on body weight. In addition, overall liking and desire to consume ratings remained stable over time, suggesting hazelnuts are resistant to monotony.

Only 9 of the 17 studies, which examined blood lipids and lipoproteins, reported between-group differences. Of these, only two studies [57,58] reported significant reductions in total and LDL-C with hazelnut consumption. Four studies also reported significantly higher HDL-C concentrations with hazelnut consumption when compared to a non-nut control [36,38,57] or a high carbohydrate diet [55]. Orem et al. also reported a significant increase in apo A [57]. Only one study reported a significant decrease in TAG [57]. The sample sizes for each treatment were small, ranging from 10 to 25. This reduces the power to detect significant differences. 

Two randomised crossover studies with larger samples (*n* = 48 to 72) compared different forms of hazelnuts and reported no significant differences in lipoprotein profiles. However, compared to baseline, hazelnut consumption significantly reduced total cholesterol, LDL-C, and apo B, and significantly increased HDL-C and apo A [43,49]. 

Several meta-analyses have reported significant improvements in blood lipids and apolipoprotein profiles with nut consumption [12,13,14]. The magnitude of the effect was greater among those with higher baseline concentrations and those with healthy body weight. There was also evidence of a dose-response relationship. In the present review, the majority of studies reported some improvement in at least one lipid parameter, with no studies reporting adverse effects. A meta-analysis of three RCTs found that hazelnut-enriched diets were associated with a reduction in total cholesterol and LDL-C, with no changes in HDL-C or TAG [31]. This suggests that similar to other nut types, hazelnuts can be incorporated into a cardioprotective diet. 

A total of 17 studies examined body composition, including body weight; BMI; waist, abdominal, and hip circumference; fat mass; and lean body mass. Except for one small single-intervention study among older adults, which showed a small but significant increase in body weight [56], the remainder of the studies among adults consistently reported no statistically or practically significant changes in body weight and composition as a result of adding hazelnuts to the diet. This is irrespective of study design, study population, study duration, and dose of hazelnuts. This was still apparent when there was no dietary advice to make substitutions. This is in agreement with a recent meta-analysis, which showed a nut-enriched diet did not result in weight gain either with or without instructions on dietary substitutions [19]. One study among children showed a time effect where there was an increase in both body weight and height. However, this did not differ between the hazelnut groups and the no-nut control. In two studies, favourable changes in body composition were seen among healthy participants [52,53]. 

These findings are consistent with other studies, which have found no evidence of weight gain in the short-term following the addition of nuts to the diet [11,19,61]. In addition, a meta-analysis of three RCTs reported no change in body weight with hazelnut consumption [31]. Possible metabolic mechanisms for this lack of weight gain include higher metabolic rate due to the high unsaturated fat content of nuts, reduced lipid bioaccessibility and higher faecal losses of lipids due to the incomplete mastication and intact cell wall of whole nuts [62]. A further possible mechanism is increased satiety, which is influenced by a number of properties found in nuts, such as the fibre and protein content, and crunchy texture, which leads to increased oral exposure time and reduced post-prandial drive for food [11,63]. Our review agrees with previous research on different nut types [11,19,24,64] and suggests that hazelnuts can be added to the diet without fear of adverse weight gain.

Seven studies measured blood pressure [39,47,49,50,53,56,59]. No significant changes to blood pressure were found as a result of adding hazelnuts to the diet. One study that included lightly salted nuts reported no significant differences in blood pressure when compared to raw, unsalted nuts. This is consistent with current literature where the effect of nut consumption on blood pressure remains equivocal, but there are suggestions of potential benefits in some sub-groups such as those with hypertension or among those without type 2 diabetes [65,66]. In addition, some nut types may be more effective, with a meta-analysis suggesting pistachios may be effective at reducing blood pressure [26,66]. The null finding is perhaps not unexpected, given the studies in the current review were conducted in relatively normotensive participants.

Nine studies measured some aspects of glycaemia, including fasting blood glucose, HbA1c, post-prandial blood glucose, fasting insulin, postprandial insulin, HOMA-IR, and iAUC for blood glucose. Consuming hazelnuts as part of a carbohydrate-rich food resulted in attenuation in blood glucose response over 2 h [40]. This has been seen in previous nut studies—including almonds [67,68] among healthy populations and pistachios among people with metabolic syndrome [69]. 

Longer-term studies in individuals with normoglycaemia showed no practical benefits from hazelnut consumption on glycaemic control. However, a single intervention among people with type 2 diabetes reported a reduction in HbAlc over 30 days [51]. It should be noted that both studies, which included people with type 2 diabetes, showed no improvements in fasting blood glucose concentrations. The mixed results are consistent with studies examining different types of nuts. Several studies have shown a lack of positive effects on glycaemia for nuts, including walnuts, almonds, and cashews [25,30,70]. In agreement with Alphan et al., a meta-analysis suggested that there may be improvements in HbA1c among people with diabetes. Collectively, the results from our review suggest that while the addition of hazelnuts to meals acutely attenuates glycaemic response, the long-term effects are less clear and require further investigation among healthy populations and those with type 2 diabetes. 

Hazelnuts are rich in antioxidants [71,72,73]. Most of the studies reported increases in antioxidant status, but this was not consistently translated into improvements of biomarkers of oxidative stress. Studies assessed different biomarkers, had relatively small samples and used different study designs and analytical methods. Previous reviews have also produced heterogenous findings [18,74], making it challenging to form definitive conclusions on the effects of nut consumption on oxidative stress. 

Six of the seven studies which examined inflammation reported no improvements. This lack of change in inflammatory markers with nut consumption, in general, was seen in previous systematic reviews and meta-analyses [75,76]. A meta-analysis of inflammatory markers, which conducted sub-group analyses, suggested improvements were seen in studies where the duration was 12 weeks and greater [17].

Five studies assessed some form of endothelial function, with three reporting improvements in outcomes and two showing no effects. Most meta-analyses on biomarkers of endothelial function report no effects with nut consumption. Those which measure flow-mediated dilation (FMD) report more favourable outcomes [76,77], especially for walnuts [16,29]. 

Overall, there was evidence that hazelnut consumption can improve some markers of cardiometabolic health. These beneficial effects are likely driven by the nutrient composition of hazelnuts. Several studies have reported improvements in diet quality with the addition of hazelnuts to the diet. There is evidence of higher intakes of unsaturated fat, fibre, vitamin E, potassium, and lower intakes of carbohydrate and sodium [43,47,49,78]. 

In addition to assessing the health effects of nut consumption, it is equally important to examine the acceptability of nuts over time. This is because to exert their health benefits, nuts must be consumed regularly and in sufficient quantities. Only one group has assessed long-term acceptance for hazelnuts. Collectively, the hazelnuts studies have reported sustained acceptance up to 12 weeks with doses of 30 to 42 g/d. One study showed a dose of 60 g/d resulted in a decline in liking with repeated consumption, a phenomenon known as monotony [47]. Future studies should assess acceptance over longer periods. Several studies examined different forms of hazelnuts, including whole, sliced, and ground, as well as raw versus dry roasted, lightly salted [40,44,48,49]. All forms of hazelnuts were resistant to monotony. A further study compared three popular energy-dense snack foods—hazelnuts, chocolate, and potato crisps. Ratings of overall liking remained stable over 12 weeks for hazelnuts but declined significantly for the other two snack foods [46]. Overall, these results suggest that dietary guidelines to consume one serving of nuts (30 to 42 g) on a regular basis are achievable and sustainable. Given that different forms of hazelnuts were equally liked, we can recommend the inclusion of different forms of nuts based on individual preference. This provides increased choice for consumers, enhancing adherence to advice to consume nuts regularly as part of a cardioprotective diet.

Studies, which have estimated the impact of substituting nuts for less healthful foods, have shown large reductions in mortality from cardiovascular disease [79,80]. In addition, a recent study reported that the total annual costs of cardiometabolic disease related to a suboptimal diet were $301 per person. Among the 10 dietary factors examined in this study, a low intake of nuts or seeds was found to impose the largest cardiometabolic disease economic burden at $81 per person [81]. Therefore, a small gradual diet change has the potential to reduce the risk of chronic disease. It seems prudent for healthcare professionals to promote the intake of healthy food such as nuts as part of a cardioprotective diet [82].

## 5. Conclusions

This comprehensive systematic review has reported the effects of hazelnut consumption on a wide range of outcomes. The findings show some improvements in cardiometabolic risk factors, but limitations in study design make interpretation difficult. However, there was consistent evidence that the inclusion of hazelnuts into the diet did not adversely affect body weight and composition. In addition, acceptance of hazelnuts remained stable over time, suggesting nut consumption guidelines are achievable and sustainable. Overall, none of the studies reported evidence of adverse outcomes, and thus the balance of the research suggests the benefits of hazelnut consumption outweigh any potential negative effects. This was apparent among populations that included healthy participants, as well as those with hyperlipidaemia, type 2 diabetes, overweight, and obesity. Future studies should use more robust study designs, including larger sample sizes, careful selection of biomarkers, and appropriate control groups. 

## Figures and Tables

**Table 1:** PICOS criteria for inclusion and exclusion of studies.

Parameter	Criterion
Participants	Humans
Intervention	Consumption of hazelnuts
Comparator	No nut control, control food, baseline
Outcomes	Total cholesterol, LDL-C, HDL-C, TAG, apolipoprotein A1, apolipoprotein B100, body weight, blood pressure, glycaemic control, antioxidant status, vitamin E, oxidative stress, inflammatory markers, endothelial function, acceptance
Study design	Intervention studies in peer-reviewed journals where hazelnuts were the dietary component under study.

**Table 2:** Study quality and risk of bias for randomised trials (*n* = 12) ^1^.

Author, Year (Study Location)	Random Sequence Generation	Allocation Concealment	Selective Reporting	Blinding	Blinding of Outcome Assessment	Incomplete Outcome Assessment	Overall Quality
Adamo et al., 2018 [36] (Italy)	low	unclear	high	high	high	low	poor
Damavandi et al., 2012 [37] (Iran)	low	unclear	low	high	low	low	good
Damavandi et al., 2013 [38] (Iran)	low	unclear	low	high	low	low	good
Deon et al., 2018 [39] (Italy)	low	unclear	low	high	low	low	good
Devi et al., 2016 [40] (New Zealand)	low	low	low	high	low	low	good
Di Renzo et al., 2017 [41] (Italy)	low	low	low	high	low	low	good
Guaraldi et al., 2018 [42] (Italy)	low	low	low	high	low	low	good
Tey et al., 2011 [43] (New Zealand)	low	low	low	high	low	low	good
Tey et al., 2011 [44] (New Zealand)	low	low	low	high	low	low	good
Tey et al., 2011 [45] (New Zealand)	low	low	low	high	low	low	good
Tey et al., 2012 [46] (New Zealand)	low	low	low	high	low	low	good
Tey et al., 2013 [47] (New Zealand)	low	low	low	high	low	low	good
Tey et al., 2015 [48] (New Zealand)	low	low	low	high	low	low	good
Tey et al., 2017 [49] (New Zealand)	low	low	low	high	low	low	good
Yilmaz et al., 2019 [50] (Turkey)	unclear	unclear	low	high	high	low	fair

^1^ Overall quality: good (low risk of bias in at least three domains), fair (low risk of bias in at least two domains), poor (low risk of bias in one or less domain). There were three studies with two publications, each reporting different study outcomes, i.e., the first study [37,38], the second study [43,44], and the third study [45,46].

**Table 3:** Study quality and risk of bias for non-randomised trials (*n* = 10) ^1^.

Author, Year (Study Location)	Bias Due to Confounding	Bias in Selection of Participants into the Study	Bias in Classification of Interventions	Bias Due to Deviations from Intended Interventions	Bias Due to Missing Data	Bias in Measurement of Outcomes	Bias in Selection of the Reported Result	Overall Risk of Bias
Alphan et al., 1997 [51] (Turkey)	critical	no information	low	low	no information	moderate	serious	critical
Di Renzo et al., 2014 [52] (Italy)	critical	low	low	low	moderate	moderate	serious	critical
Di Renzo et al., 2019 [53] (Italy)	critical	low	low	low	moderate	moderate	low	critical
Durak et al., 1999 [54] (Turkey)	critical	moderate	low	low	no information	serious	serious	critical
Mercanligil et al., 2007 [55] (Turkey)	critical	low	low	low	low	serious	low	critical
Michels et al., 2018 [56] (USA)	critical	moderate	low	low	moderate	moderate	low	critical
Orem et al., 2013 [57] (Turkey)	serious	moderate	low	low	no information	moderate	moderate	moderate
Santi et al., 2017 [58] (Italy)	serious	moderate	low	low	no information	moderate	moderate	moderate
Tey et al., 2015 [59] (New Zealand)	critical	low	low	low	moderate	moderate	low	critical
Yucesan et al., 2010 [60] (Turkey)	critical	moderate	low	low	no information	moderate	moderate	critical

^1^ Overall risk of bias judgement: low (low risk of bias for all domains), moderate (low or moderate risk of bias for all domains), serious (serious risk of bias in at least one domain, but not at critical risk of bias in any domain), critical (critical risk of bias in at least one domain).

**Table 4:** Effects of hazelnut consumption on blood lipids and lipoproteins (*n* = 17).

Author, Year	Study Design	Participant Characteristics	Duration	Treatment	TC mmol/L	LDL-C mmol/L	HDL-C mmol/L	TAG mmol/L	Between Treatments
Adamo et al., 2018 [36]	Randomised parallel 6 treatments	61 (31 M, 30 F) BMI 18.5–24.9 kg/m^2^	2-weeks	Breakfasts including: (i) 30 g/d peeled hazelnut paste	NR	NR	NR	NR	30 g of unpeeled hazelnut significantly increased HDL-C compared to control (16.0%, *p* = 0.02)
				Baseline (ii) 30 g/d unpeeled hazelnut paste	NR NR	2.49 2.33	1.68 1.82	NR NR	
				Change ^1^	NR	−0.16 ^a^	+0.14 ^b^	NR	
				% change	−2.0%	−6.0%	+16.0%	NR	
				(iii) snack with 30 g/d peeled hazelnut paste	NR	NR	NR	NR	
				(iv) snack with 2.5 g cocoa powder	NR	NR	NR	NR	
				Baseline	NR	NR	NR	NR	
				(v) Snack with 30 g/d peeled hazelnut paste and 2.5 g cocoa powder	NR	NR	NR	NR	
				% change	−0.9%	−3.4% ^a^	+5.2%	NR	
				(vi) no snack control group N.B. Data was only presented for treatment ii vs. control and treatment v vs. control	NR	NR	NR	NR	
Alphan et al., 1997 [51]	Sequential intervention periods	19 (5 M, 14 F) with type 2 diabetes	30 days	Baseline	5.40	3.36	0.95	2.78	Between-group analysis NR.
				(i) High CHO diet (60% CHO, 25% fat)	5.67	3.92	0.97	2.45	
				Change ^1^	+0.27 ^b^	+0.56 ^b^	+0.02	−0.33	
				Baseline	6.13	4.66	0.96	2.47	
				(ii) Hazelnuts (40% CHO, 45% fat—quantity of hazelnuts not reported)	5.40	3.44	1.04	2.07	
				Change ^1^	−0.73 ^b^	−1.22 ^b^	+0.08	−0.40	
Damavandi et al., 2013 [38]	Randomised parallel 2 treatments	50 (16 M, 34 F) with type 2 diabetes	8 weeks	Baseline	4.12	2.18	1.14	1.75	Significantly greater decrease in HDL-C in the control group compared to the hazelnut group (*p* = 0.009)
				(i) Hazelnuts 10% of TE	3.75	2.21	1.08	1.45	
				Change ^1^	−0.37	+0.02	−0.06	−0.30	
				Baseline	3.62	1.94	1.04	1.41	
				(ii) Control (no hazelnuts)	3.47	1.90	0.95	1.40	
				Change ^1^	−0.15	−0.04	−0.09 ^b^	−0.01	
Deon et al., 2018 [39]	Randomised parallel 3 treatments	66 children and adolescents (35 M 31 F) with hyperlipidaemia	8 weeks	Baseline	5.58	3.67	1.60	0.76 ^‡^	No significant between-group differences
				(i) Hazelnuts with skin (0.43 g /kg (15–30 g))	5.28	3.43	1.63	0.66 ^‡^	
				Change ^1^	−0.30	−0.24 ^a^	+0.03	−0.10	
				Baseline	5.73	3.66	1.58	0.69 ^‡^	
				(ii) Hazelnuts without skin (0.43 g/kg (15–30 g))	5.49	3.43	1.61	0.79 ^‡^	
				Change ^1^	−0.24	−0.23 ^a^	+0.03	+0.10	
				Baseline	5.44	3.54	1.43	0.86 ^‡^	
				Control (dietary advice only)	5.28	3.41	1.44	0.87 ^‡^	
				Change ^1^	−0.16	−0.13	+0.01	+0.01	
Di Renzo et al., 2019 [53]	Single intervention Pilot	24 (14 M, 10 F) healthy	6 weeks	Baseline	4.68 ^‡^	2.95 ^‡^	1.33 ^‡^	1.34 ^‡^	N/A, single intervention
				(i) Hazelnuts (40 g /d)	4.32 ^‡^	2.66 ^‡^	1.23 ^‡^	0.93 ^‡^	
				Change ^1^	−0.36 ^b^	−0.29 ^b^	−0.10	−0.41	
Durak et al., 1999 [54]	Single intervention	30 (18 M, 12 F) Healthy medical students	1 month	Baseline	3.38	1.95	1.03	0.86	N/A, single intervention
				(i) Hazelnuts (1 g/kg BW (68–69 g))	3.17	1.58	1.11	1.07	
				Change ^1^	−0.21 ^b^	−0.37 ^c^	+0.08 ^a^	+0.21 ^c^	
Mercanligil et al., 2007 [55]	Sequential intervention periods 2 treatments	15 (15 M, 0 F) with hyper-cholesterolaemia	4 weeks	Baseline	6.22	4.03	1.14	2.30	Compared with the control diet, the hazelnut-enriched diet significantly improved HDL-C (*p* < 0.05).
				(i) Control LF, low cholesterol, high CHO diet	5.86	3.80	1.13	2.02	
				Change ^1^	−0.36	−0.23	−0.01	−0.28	
				Baseline	6.22	4.03	1.14	2.30	
				(ii) Control + Hazelnuts (40 g)	5.89	3.90	1.28	1.57	
				Change ^1^	−0.33	−0.13	+0.14 ^a^	−0.73 ^a^	
Michels et al., 2018 [56]	Single intervention	32 (10 M, 22F F) healthy, non-frequent nut consumers, Vit E intake <10 mg a-tocopherol/d, no Vit E supplements in previous 12 months	16 weeks	Baseline	5.05	2.97	1.67	0.93	N/A, single intervention
				(i) Hazelnuts, dry roasted (~57 g/day)	4.95	2.79	1.72	0.97	
				Change ^1^	−0.1	−0.18 ^a^	+0.05	+0.04	
Orem et al., 2013 [57]	Double control sandwich model intervention	21 (18 M, 3 F) Hypercholesterolaemia	4 weeks	(i) 4 week no-nut (Control I) diet	5.77	4.01	1.12	1.65 ^‡^	Compared with the Control I period, hazelnut period significantly improved lipid and lipoprotein profile. Compared with the hazelnut period, the lipid and lipoprotein profile were significantly worse on the Control II period. All *p* < 0.05.
				(ii) 4-week hazelnut-enriched diet (49–86 g/d (18–20% TER))	5.30	3.75	1.19	1.38 ^‡^	
				Change ^1^ from (i) to (ii)	−0.47	−0.26	+0.07	−0.27	
				% change	−7.82%	−6.17%	+6.07%	−7.3%	
				(iii) 4 week no-nut (Control II) diet	5.82	4.09	1.03	1.63 ^‡^	
				Change ^1^ from (ii) to (iii)	+0.52	+0.34	−0.16	+0.25	
				% change	+9.78%	+9.37%	−3.67%	+13.7%	
Santi et al., 2017 [58]	Double control sandwich model intervention	24 (14 M, 10 F) Healthy BMI > 19 kg/m^2^, <30 kg/m^2^	6-weeks	(i) 2-week ‘standard’ diet	5.33	3.44	1.45	1.18	TC and LDL decreased significantly after the hazelnut diet compared to after Control I diet (*p =* 0.01) and *p* = 0.003, respectively). TC and LDL-C increased after Control II diet but not significantly; TC and LDL-C were significantly lower after Control II compared to after Control I i.e., the reduction during hazelnut diet remained significant (*p* = 0.04 and *p =* 0.004) respectively.
				(ii) 6-week 40 g raw hazelnut	4.90	3.08	1.38	1.20	
				Change ^1^ from (i) to (ii)	−0.43	−0.36 ^b^	−0.07	+0.02	
				(iii) 6-week ‘standard’ diet ‘washout’	5.16	3.33	1.36	1.29	
				Change ^1^ from (ii) to (iii)	+0.26	+0.25	−0.02	+0.09	
				Change ^1^ from (i) to (iii)	−0.17 ^a^	−0.11 ^b^	−0.09	+0.11	
Tey et al., 2011 [43]	Randomised Crossover 3 treatments	48 (20 M, 28 F) with mild hyper-cholesterolaemia	4 weeks	Baseline	5.88	4.01	1.21	1.43	There were no significant differences in blood lipids and lipoproteins between different forms of nuts.
				(i) Ground hazelnuts (30 g/d)	5.71	3.82	1.26	1.37	
				Change ^1^	−0.17 ^c^	−0.19 ^c^	+0.05 ^a^	−0.06	
				Baseline	5.88	4.01	1.21	1.43	
				(ii) Sliced hazelnuts (30 g/d)	5.67	3.77	1.24	1.44	
				Change ^1^	−0.21 ^c^	−0.24 ^c^	+0.03 ^a^	+0.01	
				Baseline	5.88	4.01	1.21	1.43	
				(iii) Whole hazelnuts (30 g/d)	5.63	3.74	1.25	1.39	
				Change ^1^	−0.25 ^c^	−0.27 ^c^	+0.04 ^a^	−0.04	
Tey et al., 2011 [45]	Randomised Parallel 4 treatments	118 (55 M, 63 F) Healthy, BMI < 30 kg/m^2^	12 weeks	Baseline	4.79	2.94	1.32 ^	0.98 ^	There were no significant differences in blood lipids and lipoproteins between different treatments.
				(i) Control	4.89	3.03	N/R	N/R	
				Change ^1^	+0.10	+0.09	1.00 ^	1.03 ^	
				Baseline	4.79	2.94	1.32 ^	0.98 ^	
				(ii) Hazelnuts (42 g/d)	4.73	2.85	N/R	N/R	
				Change ^1^	−0.06	−0.09	1.02 ^	0.99 ^	
				Baseline	4.79	2.94	1.32 ^	0.98 ^	
				(iii) Chocolate (50 g/d)	5.01	3.07	N/R	N/R	
				Change ^1^	+0.22	+0.13	1.04 ^	1.05 ^	
				Baseline	4.79	2.94	1.32 ^	0.98 ^	
				(iv) Potato crisp (50 g/d)	4.84	2.88	N/R	N/R	
				Change ^1^	+0.05	−0.06	1.04 ^	1.04 ^	
Tey et al., 2013 [47]	Randomised Parallel 3 treatments	107 (46 M, 61 F) BMI ≥ 25 kg/m^2^	12 weeks	Baseline	4.93	3.03	1.32	1.27	There were no significant differences in blood lipids and lipoproteins between treatments.
				(i) Control (no hazelnuts)	4.91	3.05	1.34	1.13	
				Change ^1^	−0.02	+0.02	+0.02	−0.14	
				Baseline	4.92	3.07	1.26	1.29	
				(ii) Hazelnuts (30 g/d)	4.78	2.93	1.30	1.19	
				Change ^1^	−0.14	−0.14	+0.04	−0.10	
				Baseline	4.93	3.05	1.20	1.49	
				(iii) Hazelnuts (60 g/d)	4.80	2.96	1.20	1.41	
				Change ^1^	−0.13	−0.09	0.00	−0.08	
Tey et al., 2015 [59]	Single intervention	20 Māori (8 M, 12 F) and 19 (5 M, 14 F) European aged above 18 years	4 weeks	Māori					N/A, single intervention, but there were no significant differences in blood lipids and lipoprotein between Māori and Europeans.
				Baseline	4.14^	2.46^	1.16^	1.01^	
				(i) Raw hazelnuts (30 g/d)	4.17^	2.42^	1.19^	1.04^	
				Change ^1^	N/R	N/R	N/R	N/R	
				European					
				Baseline	3.96 ^	2.28 ^	1.16 ^	0.96 ^	
				(i) Raw hazelnuts (30 g/d)	3.93 ^	2.25 ^	1.18 ^	0.94 ^	
				Change ^1^	N/R	N/R	N/R	N/R	
Tey et al., 2017 [49]	Randomised Crossover 2 treatments	72 (24 M, 48 F) Aged 18 years and above	4 weeks	Baseline	5.11	3.25	1.35	1.10	HDL-C (*p* = 0.037) was significantly higher following the consumption of raw hazelnuts, while triacylglycerol (*p* < 0.001) was significantly lower following the consumption of dry-roasted, lightly salted hazelnuts. No significant differences in TC and LDL-C between the treatments.
				(i) Raw hazelnuts (30 g/d)	5.13	3.14	1.45	1.12	
				Change ^1^	+0.02	−0.11 ^a^	+0.10 ^c^	+0.02	
				Baseline	5.11	3.25	1.35	1.10	
				(ii) Dry roasted, lightly salted hazelnuts (30 g/d)	5.06	3.17	1.41	1.03	
				Change ^1^	−0.05	−0.08	+0.06 ^c^	−0.07 ^a^	
Yilmaz et al., 2019 [50]	Randomised Parallel 4 treatments	37 (0 M, 37 F) Hyperlipidaemia, Obese	6 weeks	Baseline	6.17	4.09	1.29	1.71	There were no significant differences in blood lipids and lipoproteins between treatments.
				(i) Hazelnuts (50 g/d) and cardioprotective diet	5.61	3.61	1.36	1.40	
				Change ^1^	−0.56 ^b^	−0.48 ^b^	+0.07	−0.31	
				Baseline	6.02	3.97	1.33	1.55	
				(ii) Raisins (50 g/d) and cardioprotective diet	5.43	3.49	1.29	1.45	
				Change ^1^	−0.59 ^a^	−0.48 ^b^	−0.04	−0.10	
				Baseline	5.93	3.69	1.33	1.99	
				(iii) Hazelnuts (50 g/d) and Raisins and cardioprotective diet (50 g/d)	5.29	3.18	1.36	1.65	
				Change ^1^	−0.64 ^a^	−0.51 ^b^	+0.03	−0.34	
				Baseline	6.01	4.02	1.27	1.59	
				(iv) Control (Cardioprotective diet)	5.61	3.53	1.26	1.87	
				Change ^1^	−0.40 ^b^	−0.49 ^a^	−0.01	+0.28	
Yucesan et al., 2010 [60]	Single intervention	21 (8 M, 13 F) with normolipidaemia	4 weeks	Baseline	4.21	2.81	1.38	1.01	N/A, single intervention
				(i) Hazelnuts (1 g/kg BW (49–86 g))	3.85	2.60	1.44	0.88	
				Change ^1^	−0.36 ^c^	−0.21 ^b^	+0.06	−0.13	

To convert mmol/L TC, LDL-C, HDL-C to mg/dL, multiply mmol/L by 38.67. To convert mmol/L TAG to mg/dL, multiply mmol/L by 88.57. Abbreviations used: BW, body weight; CHO, carbohydrate; F, female; HDL-C, high-density lipoprotein cholesterol; LDL-C, low-density lipoprotein cholesterol; LF, low fat; M, male; N/A, not applicable; NR, not reported; TAG, triacylglycerols; TC, total-cholesterol, TER, total energy requirement. All values are arithmetic means unless otherwise stated. ^1^ Change (within-group) = Post-treatment value minus Pre-treatment value (i.e., baseline); ^a^ *p* < 0.05; ^b^ *p* < 0.01; ^c^ *p* < 0.001; only for those which reported within-group change. ^ Geometric mean, and differences are ratios of the geometric means; ^‡^ Median.

**Table 5:** Effects of hazelnut consumption on apolipoprotein A1 and B100 (*n* = 8).

Author, Year	Study Design	Participant Characteristics	Duration	Treatment	Apo A g/L	Apo B g/L	Between Treatments
Alphan et al., 1997 [51]	Sequential intervention periods 2 treatments	19 (5 M, 14 F) Type 2 diabetics	30 days	Baseline	1.89	2.29	Between-group analysis NR.
				(i) High CHO diet (60% CHO, 25% fat)	2.33	2.92	
				Change ^1^	+0.44	+0.63	
				Baseline	1.81	2.03	
				(ii) Hazelnuts (40% CHO, 45% fat, hazelnuts amount NR)	1.94	1.87	
				Change ^1^	+0.13	−0.16	
Mercanligil et al., 2007 [55]	Sequential intervention periods 2 treatments	15 (15 M, 0 F) Hyper-cholesterolaemic	4 weeks	Baseline	1.36	1.33	There were no significant differences in apo A and apo B between the diets.
				(i) Control LF, low cholesterol, high CHO diet	1.32	1.28	
				Change ^1^	−0.04	−0.05	
				Baseline	1.36	1.33	
				(ii) Control + Hazelnuts (40 g/d)	1.36	1.21	
				Change ^1^	0.00	−0.12 ^a^	
Orem et al., 2013 [57]	Double control sandwich model intervention	21 (18 M, 3 F) Hyper-cholesterolaemic	4 weeks	(i) 4 week no-nut (Control I) diet	1.31	1.15	Apo A significantly increased after hazelnut period compared to Control I. Apo A significantly decreased after the Control II period compared to the hazelnut-enriched diet Apo B significantly increased after the Control II period compared to the hazelnut-enriched diet
				(ii) 4-week hazelnut-enriched diet (49–86 g/d (18–20% TER))	1.46	1.12	
				(iii) 4 week no-nut (Control II) diet	1.38	1.20	
				% change from (i) to (ii)	+12.0	−1.90	
				% change from (ii) to (iii)	−5.61	+15.2	
Tey et al., 2011 [43]	Randomised Crossover 3 treatments	48 (20 M, 28 F) Mildly hyper-cholesterolaemic	4 weeks	Baseline	1.78	1.05	There were no significant differences in apo A and apo B between the different forms of nuts.
				(i) Ground hazelnuts (30 g)	1.79	1.02	
				Change ^1^	+0.01	−0.03 ^b^	
				Baseline	1.78	1.05	
				(ii) Sliced hazelnuts (30 g)	1.78	1.01	
				Change 1	0.00	−0.04 ^b^	
				Baseline	1.78	1.05	
				(iii) Whole hazelnuts (30 g)	1.79	1.00	
				Change ^1^	+0.01	−0.05 ^b^	
Tey et al., 2013 [47]	Randomised Parallel 3 treatments	107 (46 M, 61 F) Overweight and obese individuals with a BMI ≥ 25 kg/m^2^	12 weeks	Baseline	1.67	0.87	There were no significant differences in apo A and apo B between the groups.
				(i) Control group (no hazelnuts)	1.65	0.86	
				Change ^1^	−0.02	−0.01	
				Baseline	1.60	0.88	
				(ii) Hazelnuts (30 g/d)	1.63	0.85	
				Change ^1^	+0.03	−0.03	
				Baseline	1.56	0.89	
				(ii) Hazelnuts (60 g/d)	1.57	0.87	
				Change ^1^	+0.01	−0.02	
Tey et al., 2015 [59]	Single intervention	20 Māori (8 M, 12 F) and 19 (5 M, 14 F) European aged above 18 years	4 weeks	Māori			N/A, single intervention, but there were no significant differences in apo A and apo B between Māori and Europeans.
				Baseline	1.51 ^	0.71 ^	
				(i) Raw hazelnuts (30 g/d)	1.57 ^	0.70 ^	
				Change ^1^	N/R	N/R	
				European			
				Baseline	1.51 ^	0.65 ^	
				(i) Raw hazelnuts (30 g/d)	1.52 ^	0.63 ^	
				Change ^1^	N/R	N/R	
Tey et al., 2017 [49]	Randomised Crossover 2 treatments	72 (24 M, 48 F) Aged 18 years and above	4 weeks	Baseline	1.59	0.87	There were no significant differences in apo A and apo B between the groups.
				(i) Raw hazelnuts (30 g/d)	1.65	0.86	
				Change ^1^	+0.06 ^b^	−0.01	
				Baseline	1.59	0.87	
				(ii) Dry roasted, lightly salted hazelnuts (30 g/d)	1.63	0.86	
				Change ^1^	+0.04 ^b^	−0.01	
Yucesan et al., 2010 [60]	Single intervention	21 (8 M, 13 F) Normolipidaemic	4 weeks	Baseline	1.35	0.78	N/A, single intervention
				(i) Hazelnuts (1 g/kg BW (49–86 g))	1.41	0.71	
				Change ^1^	+0.06 ^b^	−0.07 ^b^	

Abbreviations used: apo, apolipoprotein; BW, body weight; CHO, carbohydrate; F, female; LF, low fat; M, male; N/A, not applicable; NR, not reported; TE, total energy; TER, total energy requirement. All values are arithmetic means unless otherwise stated. ^1^ Change (within-group) = Post-treatment value minus Pre-treatment value (i.e., baseline); ^a^ *p <* 0.05; ^b^ *p <* 0.01; only for those which reported within-group change. ^ Geometric mean.

**Table 6:** Effects of hazelnut consumption on body weight (*n* = 17).

Author, Year	Study Design	Participant Characteristics	Duration	Comparison Made Body Weight	Treatment Body Weight	Change in Body Weight ^1^	Between Treatments
Alphan et al., 1997 [51]	Sequential intervention periods 2 treatments	19 (5 M, 14 F) Type 2 diabetics	30 days	(i) Baseline BMI: 27.5 kg/m^2^	(i) High CHO diet (60% CHO, 25% fat) BMI: 27.3 kg/m^2^	(i) No significant change	Between-group analysis NR.
				(ii) Baseline BMI: 27.1 kg/m^2^	(ii) Hazelnut diet (40% CHO, 40% fat, Hazelnut amount NR) BMI: 27.1 kg/m^2^	(ii) No significant change	
Damavandi et al., 2012 [37]	Randomised parallel 2 treatments	50 (16 M, 34 F) with type 2 diabetes	8 weeks	(i)Baseline Weight: 72.13 kg BMI: 28.47 kg/m^2^	(i) Hazelnut (10% TE) Weight: 71.47 kg BMI: 27.92 kg/m^2^	(i) No significant change	No significant between-group differences in body weight or BMI.
				(ii) Baseline Weight: 71.98 kg BMI: 28.18 kg/m^2^	(ii) Control: no hazelnuts Weight: 71.64 kg BMI: 28.08 kg/m^2^	(ii) No significant change	
Deon et al., 2018 [39]	Randomised parallel 3 treatments	66 children and adolescents (35 M 31 F) with hyperlipidaemia	8 weeks	(i) Baseline Weight: 44.4 kg BMI: 20.4 kg/m^2^	(i) Hazelnuts with skin (0.43 g/kg (15–30 g/d)) Weight: 45.0 kg BMI: 20.3 kg/m^2^	(i) No significant change for BMI	No significant between-group differences in BMI, there was a time effect for height and weight.
				(ii) Baseline Weight: 47.8 kg BMI: 20.3 kg/m^2^	(ii) Hazelnuts without skin (0.43 g/kg (15–30 g/d)) Weight: 48.4 kg BMI: 20.3 kg/m^2^	(ii) No significant change for BMI	
				(iii) Baseline Weight: 49.5 kg BMI: 20.9 kg/m^2^	(iii) Control: no hazelnuts Weight: 50.0 kg BMI: 20.8 kg/m^2^	(iii) No significant change for BMI	
Di Renzo et al., 2014 [52]	Sequential intervention periods 2 treatments	24 participants BMI ≥ 19 kg/m^2^	4 weeks	(i) Baseline Weight: 66.15 kg	(i) 4 week standard diet (Italian Mediterranean diet) Weight: 67.8 kg WC: 77.44 cm HC: 97.5 cm Fat mass: 16.93 kg LBM: 34.56 kg	NR	HC and LBM was significantly higher, and fat mass was significantly lower after the hazelnut diet compared to the standard diet (all *p* < 0.05).
				(ii) Baseline NR	(ii) Hazelnuts (40 g/d) Weight: 66.8 kg WC: 76.43 cm HC: 99.76 cm Fat mass: 14.83 kg LBM: 35.07 kg		
Di Renzo et al., 2019 [53]	Single intervention Pilot	24 (14 M, 10 F) healthy	6 weeks	(i) Baseline ^‡^ Weight: 71.4 kg BMI: 25.95 kg/m^2^ WC: 86.25 cm AC: 94.00 cm HC: 98.25 cm Total body fat: 34.75 kg Total BF: 29.65% Android BF: 28.75% Gynoid BF: 21.08% LBM: 47.63 kg ASMMI: 8.37	(i) Hazelnuts (40 g/d) ^‡^ Weight: 71.05 kg BMI: 25.76 kg/m^2^ WC: 85.00 cm AC: 93.50 cm HC: 99.00 cm Total body fat: 34.95 kg Total BF: 29.05% Android BF: 28.80% Gynoid BF: 21.34% LBM: 48.09 kg ASMMI: 8.05	(i) AC was significantly lower after the hazelnut intervention	N/A, single intervention
Durak et al., 1999 [54]	Single intervention	30 (18 M, 12 F) Healthy Medical students	1 month	(i) Habitual diet Weight: 68.7 kg	(i) Hazelnuts (1 g/kg BW (68–69 g)) Weight: 69.2 kg	(i) No significant change	N/A, single intervention
Mercanligil et al., 2007 [55]	Sequential intervention periods 2 treatments	15 (15 M, 0 F) Hyper-cholesterolaemic	4 weeks	(i) Baseline Weight: 74.3 kg	(i) Control LF, high CHO diet Weight: 74.2 kg	(i) No significant change	No significant between-group differences in body weight.
				(ii) Baseline Weight: 74.3 kg	(ii) Control + Hazelnuts (40 g/d) Weight: 74.0 kg	(ii) No significant change	
Michels et al., 2018 [56]	Single intervention	32 (10 M, 22F F) healthy, non-frequent nut consumers, Vit E intake <10 mg a-tocopherol/d, no Vit E supplements in previous 12 months	16 weeks	(i) Baseline BMI: 26.1 kg/m^2^	(i) Hazelnuts, dry roasted (~57 g/day) BMI: 26.3 kg/m^2^	(i) BMI: +0.2 kg/m^2^ (*p* = 0.009)	N/A, single intervention
Orem et al., 2013 [57]	Double control sandwich model intervention	21 (18 M, 3 F) Hyper-cholesterolaemic	4 weeks	(i) 4 week no-nut (Control I) diet Weight: 81.0 kg BMI: 27.4 kg/m^2^	(ii) 4-week hazelnut-enriched diet (49–86 g/d (18–20% TER)) Weight: 79.1 kg BMI: 26.9 kg/m^2^	(i) to (ii): Weight: −0.9 kg; −2.3% BMI: −0.5 kg/m^2^; −2.02%	Body weight and BMI were significantly different between (i) and (ii) and between (i) and (iii). There was no significant difference in body weight or BMI between (ii) and (iii).
				(ii) 4-week hazelnut-enriched diet (49–86 g/d (18–20% TER)) Weight: 79.1 kg BMI: 26.9 kg/m^2^	(iii) 4 week no-nut (Control II) diet Weight: 79.5 kg BMI: 26.9 kg/m^2^	(ii) to (iii): Weight: +0.4 kg; +0.4% BMI: no numerical change; +0.07%	
Santi et al., 2017 [58]	Double control sandwich model intervention	24 (14 M, 10 F) Healthy, BMI > 19 kg/m^2^, <30 kg/m^2^	6-weeks	(i) 2-week ‘standard’ diet	(ii) 6-week raw hazelnut (40 g/d) (iii) 6-week ‘standard’ diet ‘washout’	No significant changes in body weight	NR
Tey et al., 2011 [43]	Randomised Crossover 3 treatments	48 (20 M, 28 F) Mildly hyper-cholesterolaemic	4 weeks	(i) Baseline Weight: 73.7 kg BMI: 25.7 kg/m^2^	(i) Ground hazelnuts (30 g/d) Weight: 73.8 kg BMI: 25.8 kg/m^2^	(i) No significant change	No significant between-group differences in body weight or BMI.
				(ii) Baseline Weight: 73.7 kg BMI: 25.7 kg/m^2^	(ii) Sliced hazelnuts (30 g/d) Weight: 74.0 kg BMI: 25.9 kg/m^2^	(ii) No significant change	
				(iii) Baseline Weight: 73.7 kg BMI: 25.7 kg/m^2^	(iii) Whole hazelnuts (30 g/d) Weight: 74.0 kg BMI: 25.9 kg/m^2^	(iii) No significant change	
Tey et al., 2011 [45]	Randomised Parallel 4 treatments	118 (55 M, 63 F) Healthy, BMI < 30 kg/m^2^	12 weeks	(i) Baseline Weight: 67.3 kg BMI: 22.9 kg/m^2^ Body fat: 25.8% Waist circ: 79.0 cm	(i) Control (no hazelnuts) Weight: 67.76 kg BMI: 23.04 kg/m^2^ Body fat: 24.96% Waist circ: 80.36 cm	(i) No significant change	No significant between-group differences in body weight, BMI, body fat, and waist circumference.
				(ii) Baseline Weight: 72.0 kg BMI: 24.6 kg/m^2^ Body fat: 28.1% Waist circ: 82.1 cm	(ii) Hazelnuts (42 g/d) Weight: 72.83 kg BMI: 24.88 kg/m^2^ Body fat: 27.35% Waist circ: 84.23 cm	(ii) No significant change	
				(iii) Baseline Weight: 69.2 kg BMI: 23.6 kg/m^2^ Body fat: 26.7% Waist circ: 80.2 cm	(iii) Chocolate (50 g/d) Weight: 69.79 kg BMI: 23.81 kg/m^2^ Body fat: 25.47% Waist circ: 81.5 cm	(iii) No significant change	
				(iv) Baseline Weight: 69.5 kg BMI: 23.9 kg/m^2^ Body fat: 26.9% Waist circ: 81.7 cm	(iv) Potato crisps (50 g/d) Weight: 70.0 kg BMI: 24.05 kg/m^2^ Body fat: 25.81% Waist circ: 81.17 cm	(iv) No significant change	
Tey et al., 2013 [47]	Randomised Parallel 3 treatments	107 (46 M, 61 F) Overweight and obese individuals with a BMI ≥ 25 kg/m^2^	12 weeks	(i) Baseline Weight: 88.7 kg BMI: 30.4 kg/m^2^ Body fat: 33.9% Fat mass: 30.1 kg Fat-free mass: 58.7 kg	(i) Control (no hazelnuts) Weight: 88.7 kg BMI: 30.4 kg/m^2^ Body fat: 33.9% Fat mass: 30.1 kg Fat-free mass: 58.6 kg	(i) No significant change	There were no significant differences in body weight, BMI, body fat percent, fat mass, and fat-free mass between the treatments.
				(ii) Baseline Weight: 86.2 kg BMI: 30.7 kg/m^2^ Body fat: 35.4% Fat mass: 30.7 kg Fat-free mass: 55.5 kg	(ii) Hazelnuts (30 g/d) Weight: 86.2 kg BMI: 30.7 kg/m^2^ Body fat: 35.4% Fat mass: 30.7 kg Fat-free mass: 55.6 kg	(ii) No significant change	
				(iii) Baseline Weight: 92.0 kg BMI: 30.9 kg/m^2^ Body fat: 35.0% Fat mass: 32.5 kg Fat-free mass: 59.5 kg	(iii) Hazelnuts (60 g/d) Weight: 92.2 kg BMI: 30.9 kg/m^2^ Body fat: 34.9% Fat mass: 32.5 kg Fat-free mass: 59.7 kg	(iii) No significant change	
Tey et al., 2015 [59]	Single intervention	20 Māori (8 M, 12 F) and 19 (5 M, 14 F) European aged above 18 years	4 weeks	Māori (i) Baseline Weight ^: 76.3 kg BMI ^: 25.5 kg/m^2^ Body fat ^: 26.9%	Māori (i) Hazelnuts (30 g/d) Weight ^: 76.3 kg BMI ^: 25.5 kg/m^2^ Body fat ^: 27.3%	Māori (i) No significant change	N/A, single intervention, but there were no significant differences in body weight, BMI, and body fat percent between Māori and Europeans.
				European (ii) Baseline Weight ^: 71.5 kg BMI ^: 24.4 kg/m^2^ Body fat ^: 25.9%	European (ii) Hazelnuts (30 g/d) Weight ^: 71.8 kg BMI ^: 24.4 kg/m^2^ Body fat ^: 26.6%	European (i) No significant change	
Tey et al., 2017 [49]	Randomised Crossover 2 treatments	72 (24 M, 48 F) Aged 18 years and above	4 weeks	(i) Baseline Weight: 76.7 kg BMI: 26.7 kg/m^2^ Body fat: 32.0% Fat mass: 25.1 kg Fat-free mass: 51.6 kg	(i) Raw hazelnuts (30 g/d) Weight: 76.57 kg BMI: 26.65 kg/m^2^ Body fat: 31.83% Fat mass: 24.83 kg Fat-free mass: 51.71 kg	(i) No significant change	There were no significant differences in body weight, BMI, body fat, fat mass, and fat-free mass between the treatments.
				(ii) Baseline Weight: 76.7 kg BMI: 26.7 kg/m^2^ Body fat: 32.0% Fat mass: 25.1 kg Fat-free mass: 51.6 kg	(ii) Dry roasted, lightly salted hazelnuts (30 g/d) Weight: 76.67 kg BMI: 26.68 kg/m^2^ Body fat: 31.86% Fat mass: 24.96 kg Fat-free mass: 51.69 kg	(ii) No significant change	
Yilmaz et al., 2019 [50]	Randomised Parallel 4 treatments	37 (0 M, 37 F) Hyperlipidaemia, Obese	6 weeks	(i) Baseline Weight: 78.5 kg BMI: 35.7 kg/m^2^ Waist circ: 104.1 cm Waist/Hip ratio: 0.88 Fat mass: 34.0 kg Fat mass: 43.1%	(i) Hazelnuts (50 g/d) and cardioprotective diet Weight: 76.0 kg BMI: 34.5 kg/m^2^ Waist circ: 98.3 cm Waist/Hip ratio: 0.85 Fat mass: 31.8 kg Fat mass: 41.7%	(i) Hazelnuts (50 g/d) Weight: −2.5 kg (*p* = 0.030) BMI: −1.2 kg/m^2^ (*p* = 0.031) Waist circ: −5.7 cm (*p* = 0.113) Waist/Hip ratio: −0.03 (*p* = 0.650) Fat mass: −2.21 kg (*p* = 0.005) Fat mass: −1.41% (*p* = 0.001)	There were no significant differences in body weight, BMI, waist circumference, waist/hip ratio, and fat mass between the treatments.
				(ii) Baseline Weight: 83.7 kg BMI: 35.8 kg/m^2^ Waist circ: 106.1 cm Waist/Hip ratio: 0.89 Fat mass: 35.4 kg Fat mass: 41.9%	(ii) Raisins (50 g/d) and cardioprotective diet Weight: 82.2 kg BMI: 35.1 kg/m^2^ Waist circ: 101.2 cm Waist/Hip ratio: 0.86 Fat mass: 34.1 kg Fat mass: 41.1%	(ii) Raisins (50 g/d) Weight: −1.5 kg (*p* = 0.074) BMI: −0.7 kg/m^2^ (*p* = 0.046) Waist circ: −4.9 cm (*p* = 0.0001) Waist/Hip ratio: −0.03 (*p* = 0.009) Fat mass: −1.32 kg (*p* = 0.021) Fat mass: −0.90% (*p* = 0.241)	
				(iii) Baseline Weight: 80.0 kg BMI: 34.6 kg/m^2^ Waist circ: 98.3 cm Waist/Hip ratio: 0.85 Fat mass: 33.3 kg Fat mass: 41.4%	(iii) Hazelnuts (50 g/d) and Raisins (50 g/d) and cardioprotective diet Weight: 77.9 kg BMI: 33.6 kg/m^2^ Waist circ: 95.1 cm Waist/Hip ratio: 0.85 Fat mass: 31.1 kg Fat mass: 39.6%	(iii) Hazelnuts (50 g/d) and Raisins (50 g/d) Weight: −2.1 kg (*p* = 0.002) BMI: −0.9 kg/m^2^ (*p* = 0.004) Waist circ: −3.2 cm (*p* = 0.122) Waist/Hip ratio: −0.01 (*p* = 1.000) Fat mass: −2.26 kg (*p* = 0.001) Fat mass: −1.72% (*p* = 0.002)	
				(iv) Baseline Weight: 81.9 kg BMI: 36.0 kg/m^2^ Waist circ: 108.1 cm Waist/Hip ratio: 0.91 Fat mass: 35.7 kg Fat mass: 43.4%	(iv) Control (Cardioprotective diet) Weight: 79.6 kg BMI: 34.9 kg/m^2^ Waist circ: 99.9 cm Waist/Hip ratio: 0.87 Fat mass: 33.5 kg Fat mass: 41.9%	(iv) Control (Cardioprotective diet) Weight: −2.4 kg (*p* = 0.017) BMI: −1.1 kg/m^2^ (*p* = 0.020) Waist circ: −8.2 cm (*p* = 0.002) Waist/Hip ratio: −0.05 (*p* = 0.009) Fat mass: −2.17 kg (*p* = 0.002) Fat mass: −1.42% (*p* = 0.003)	
Yucesan et al., 2010 [60]	Single intervention	21 (8 M, 13 F) Normolipidaemic	4 weeks	(i) Baseline: 64.5 kg	(i) Hazelnuts (1 g/kg BW (49–86 g/d)): 64.7 kg	(i) No significant change	N/A, single intervention

Abbreviations used: AC, abdominal circumference; ASMMI: appendicular skeletal muscle mass index; BF, body fat; BMI, body mass index; BW, body weight; CHO, carbohydrate; circ, circumference; F, female; HC, hip circumference; LBM, lean body mass; LF, low fat; M, male; N/A, not applicable; NR, not reported; TE, total energy; TER, total energy requirement; WC, waist circumference. All values are arithmetic means unless otherwise stated. ^1^ Change (within-group) = Post-treatment value minus Pre-treatment value (i.e., baseline). ^ Geometric mean. ^‡^ Median.

**Table 7:** Effects of hazelnut consumption on blood pressure (*n* = 7).

Author, Year	Study Design	Participant Characteristics	Duration	Treatment	SBP mmHg	DBP mmHg	Between Treatments
Deon et al., 2018 [39]	Randomised parallel 3 treatments	66 children and adolescents (35 M 31 F) with hyperlipidaemia	8 weeks	(i) Baseline	103.0	65.6	No significant differences in systolic blood pressure or diastolic blood pressure between the treatments.
				Hazelnuts with skin (0.43 g/kg (15–30 g/d))	105.2	66.4	
				Change ^1^	+2.2	+0.8	
				(ii) Baseline	102.8	65.1	
				Hazelnuts without skin (0.43 g/kg (15–30 g/d))	102.5	66.3	
				Change ^1^	−0.3	+1.2	
				(iii) Baseline	106.8	68.0	
				Control	109.0	67.1	
				Change ^1^	+2.2	−0.9	
Di Renzo et al., 2019 [53]	Single intervention Pilot	24 (14 M, 10 F) healthy	6 weeks	Baseline ^‡^	116.5	73.0	N/A, single intervention.
				(i) Hazelnuts (40 g/d) ^‡^	112.0	75.0	
				Change ^1^	−4.5	+2.0	
Michels et al., 2018 [56]	Single intervention	32 (10 M, 22 F) healthy, non-frequent nut consumers, Vit E intake <10 mg a-tocopherol/d, no Vit E supplements in previous 12 months	16 weeks	Baseline	120	76.6	N/A, single intervention.
				(i) Hazelnuts, dry roasted (~57 g/d)	120	76.3	
				Change ^1^	0	−0.3	
Tey et al., 2013 [47]	Randomised Parallel 2 treatments	107 (46 M, 61 F) Overweight and obese individuals with a BMI ≥ 25 kg/m^2^	12 weeks	Baseline	128	75.3	No significant difference in systolic and diastolic blood pressure between the treatments.
				(i) Control (no hazelnuts)	123	72.9	
				Change ^1^	−5 ^a^	−2.4 ^a^	
				Baseline	126	73.2	
				(ii) Hazelnuts (30 g/d)	124	72.6	
				Change ^1^	−2	−0.6	
				Baseline	124	76.3	
				(iii) Hazelnuts (60 g/d)	121	73.3	
				Change ^1^	−3 ^a^	−3.0 ^a^	
Tey et al., 2015 [59]	Single intervention	20 Māori (8 M, 12 F) and 19 (5 M, 14 F) European aged above 18 years	4 weeks	Māori Baseline ^ (i) Raw hazelnuts (30 g/d) ^ Change ^1^	123.6 117.1 N/R	67.7 68.5 N/R	N/A, single intervention, but there were no significant differences in systolic and diastolic blood pressure between Māori and Europeans.
				European Baseline ^ (i) Raw hazelnuts (30 g/d) ^ Change ^1^	120.1 118.4 N/R	65.5 65.1 N/R	
Tey et al., 2017 [49]	Randomised Crossover 2 treatments	72 (24 M, 48 F) Aged 18 years and above	4 weeks	Baseline	124	73.5	No significant differences in systolic blood pressure between the treatments. There was a tendency that diastolic blood pressure was lower after consuming dry roasted and lightly salted hazelnuts.
				(i) Raw hazelnuts (30 g/d)	122	72.7	
				Change ^1^	−2.0 ^a^	−0.8	
				Baseline	124	73.5	
				(ii) Dry roasted, lightly salted hazelnuts (30 g/d)	121.1	71.5	
				Change ^1^	−2.9 ^b^	−2.0 ^b^	
Yilmaz et al., 2019 [50]	Randomised Parallel 4 treatments	37 (0 M, 37 F) Hyperlipidaemia, Obese	6 weeks	Baseline	121.7	77.2	No significant difference in systolic and diastolic blood pressure between the treatments.
				(i) Hazelnuts (50 g/d) and cardioprotective diet	121.1	75.6	
				Change ^1^	−0.6	−1.7	
				Baseline	123.3	76.7	
				(ii) Raisins (50 g/d) and cardioprotective diet	119.4	76.7	
				Change ^1^	−3.9	0.0	
				Baseline	123.6	79.7	
				(iii) Hazelnuts (50 g/d) and Raisins (50 g/d) and cardioprotective diet	115.6	75.6	
				Change ^1^	−8.0 ^a^	−4.1	
				Baseline	126.0	80.5	
				(iv) Control (Cardioprotective diet)	122.0	77.5	
				Change ^1^	−4.0	−3.0	

Abbreviations used: DBP, diastolic blood pressure; F, female; M, male; N/A, not applicable; SBP, systolic blood pressure. All values are arithmetic means unless otherwise stated. ^1^ Change (within-group) = Post-treatment value minus Pre-treatment value (i.e., baseline); ^a^ *p <* 0.05; ^b^ *p <* 0.01; only for those which reported within-group change. ^ Geometric mean. ^‡^ Median.

**Table 8:** Effects of hazelnut consumption on glycaemic outcomes (*n* = 9).

Author, Year	Study Design	Participant Characteristics	Duration	Treatment	Outcome Measurements: Results
Acute study					
Devi et al., 2016 [40]	Randomised crossover 4 treatments	32 (11 M 21 F) healthy	Acute 2 h	(i) Bread containing 30 g finely sliced hazelnuts per 120 g	2 h iAUC for blood glucose (i) 152 mmol/L·min
				(ii) Bread containing 30 g defatted hazelnut flour per 120 g	(ii) 137 mmol/L·min
				(iii) Bread containing 15 g finely sliced hazelnuts and 15 g defatted hazelnut flour per 120 g	(iii) 154 mmol/L·min
				(iv) Control white bread with no nuts	(iv) 179 mmol/L·min All hazelnut breads had a lower iAUC compared to the control bread (all *p* < 0.001). There were no significant differences between breads.
Chronic studies					
Adamo et al., 2017 [36]	Randomised parallel 6 treatments	61 (31 M, 30 F) Healthy BMI	2 weeks	Breakfasts including: (i) 30 g peeled hazelnut paste	Insulin and HOMA-IR Insulin and HOMA-IR remained stable in those consuming the hazelnut-only enriched breakfasts. Actual data was not presented.
				(ii) 30 g unpeeled hazelnut paste	
				(iii) snack with 30 g peeled hazelnut paste	
				(iv) snack with 2.5 g cocoa powder	
				(v) Snack with 30 g/d peeled hazelnut paste and 2.5 g cocoa powder	
				(vi) no snack control group *N.B. Data was only presented for treatments* vs. *control i.e., no other between-group comparisons were reported*	
Alphan et al., 1997 [51]	Sequential intervention periods 2 treatments	19 (5 M, 14 F) Type 2 diabetics	30 days	(i) High CHO diet (60% CHO, 25% fat): HbA1c Baseline: 8.1% End: 7.8% Change: −0.3% FBG Baseline6.92 mmol/L End: 6.94 mmol/L Change: +0.02 mmol/L PPBG Baseline9.16 mmol/L End: 8.49 mmol/L Change: −0.67 mmol/L Fasting insulin Baseline: 86.4 pmol/L End: 72.6 pmol/L Change: −13.4 pmol/L PP insulin Baseline 249.0 pmol/L End: 196.8 pmol/L Change: −52.2 pmol/L	Between-group analysis NR.
				(ii) Hazelnut diet (40% CHO, 45% fat, amount of hazelnuts NR): HbA1c Baseline: 8.3% End: 7.2% Change: −1.1% ^a^ FBG: Baseline: 7.28 mmol/L End: 7.28 mmol/L Change: 0.00 mmol/L PPBG Baseline: 8.37 mmol/L End:8.28 mmol/L Change: −0.09 mmol/L Fasting insulin Baseline: 78.0 pmol/L End:97.2 pmol/L Change: +19.2 pmol/L PP insulin Baseline: 223.2 pmol/L End: 225.0 pmol/L Change: +1.8 pmol/L	
Damavandi et al., 2012 [37]	Randomised parallel 2 treatments	50 (16 M, 34 F) participants with type 2 diabetes	8 weeks	(i) Control: No hazelnuts FBG Baseline: 8.69 mmol/L End: 8.97 mmol/L Change: +0.28 mmol/L	Fasting blood glucose There were no significant differences in fasting blood glucose concentrations
				(ii) 10% of total energy hazelnuts FBG Baseline: 8.10 mmol/L End: 8.04 mmol/L Change: −0.06 mmol/L	
Michels et al., 2018 [56]	Single intervention	32 (10 M, 22F F) healthy, non-frequent nut consumers, Vit E intake <10 mg a-tocopherol/d, no Vit E supplements in previous 12 months	16 weeks	(i) Baseline FBG: 5.67 mmol/L Fasting insulin: 48.6 pmol/L	Significant reduction in plasma FBG (−3.4%, *p* = 0.03) after 16 weeks consuming 57 g/day hazelnuts. There was no significant change in fasting insulin.
				(ii) Hazelnuts, dry roasted (~57 g/day) FBG: 5.5 mmol/L Fasting insulin: 49.8 pmol/L	
Orem et al., 2013 [57]	Double control sandwich model intervention	21 (18 M, 3 F) Hyper-cholesterolaemic	4 weeks	(i) 4 week no-nut (Control I) diet FBG: 5.22 mmol/L Fasting insulin: 42.6 pmol/L HOMA-IR: 1.69	There was no significant difference in FBG, fasting insulin, or HOMA-IR between treatments.
				(ii) 4-week hazelnut-enriched diet (49–86 g/d (18–20% TER)) FBG: 5.11 mmol/L, Δ: −1.52% Fasting insulin: 45.6 pmol/L, Δ: +14.7% HOMA-IR: 1.78, Δ: +13.1%	
				(iii) 4-week no-nut (Control II) diet FBG: 4.89 mmol/L, Δ: −3.51% Fasting insulin: 37.8 pmol/L, Δ: −11.9% HOMA-IR: 1.39, Δ: −12.7%	
Santi et al., 2017 [58]	Double control sandwich model intervention	24 (14 M, 10 F) Healthy BMI > 19 kg/m^2^, <30 kg/m^2^	6 weeks	(i) 2-week ‘standard’ diet FBG: 4.79 mmol/L	There was no significant difference in FBG between treatments.
				(ii) 6-week 40 g raw hazelnut FBG: 4.76 mmol/L	
				(iii) 6-week ‘standard’ diet ‘washout’ FBG: 4.77 mmol/L	
Tey et al., 2017 [49]	Randomised Crossover 2 treatments	72 (24 M, 48 F) Aged 18 years and above	4 weeks	Fasting blood glucose Baseline: 4.82 mmol/L (i) Raw hazelnuts (30 g/d): 4.80 mmol/L Change: −0.02 mmol/L	There was no significant difference in fasting blood glucose between the treatments.
				Baseline: 4.82 mmol/L (ii) Dry roasted, lightly salted hazelnuts (30 g/d): 4.81 mmol/L Change: −0.01 mmol/L	
Yilmaz et al., 2019 [50]	Randomised Parallel 4 treatments	37 (0 M, 37 F) Hyperlipidaemia, Obese	6 weeks	Fasting blood glucose Baseline: 5.23 mmol/L (i) Hazelnuts (50 g/d): 5.18 mmol/L Change: −0.05 mmol/L	There was no significant difference in fasting blood glucose between the treatments.
				Baseline: 5.16 mmol/L (ii) Raisins (50 g/d): 5.64 mmol/L Change: +0.48 mmol/L	
				Baseline: 5.33 mmol/L (iii) Hazelnuts (50 g/d) and Raisins (50 g/d): 5.17 mmol/L Change: −0.16 mmol/L	
				Baseline: 5.26 mmol/L (iv) Control (Cardioprotective diet): 5.47 mmol/L Change: +0.21 mmol/L	

To convert mmol/L blood glucose to mg/dL, multiply mmol/L by 18. Abbreviations used: F, female; FBG, fasting blood glucose; HbA1c, glycated haemoglobin; HOMA-IR, homeostasis model-insulin resistance; iAUC, incremental area under the curve; M, male; NR, not reported; PP, postprandial; PPBG, postprandial blood glucose; TER, total energy requirement. All values are arithmetic means unless otherwise stated. ^a^ *p <* 0.05 only for those which reported within-group change.

**Table 9:** Effects of hazelnut consumption on antioxidant, oxidative stress, inflammatory markers, and endothelial function (*n* = 16).

Author, Year	Study Design	Participant Characteristics	Duration	Treatments	Outcome Measurements: Results ^1^
Acute study					
Di Renzo et al., 2017 [41]	Randomised crossover 2 treatments	22 healthy BMI ≥ 19 kg/m^2^ BMI < 30 kg/m^2^	3 h	(i) A high-fat McDonald’s meal	Oxidised LDL using ELISA kits (i) Levels increased significantly by 18% from fasting to after the McDonald’s meal ^a^ (ii) No significant difference in levels for the McDonald’s meal with 40 g of hazelnuts Levels were significantly lower after the McDonald’s meal with 40 g of hazelnuts compared to the McDonald’s meal (−24.43%, *p* < 0.05) N.B. Actual baseline and end of study values NR
				(ii) A high-fat McDonald’s meal with 40 g of hazelnuts	
Chronic studies					
Adamo et al., 2017 [36]	Randomised parallel 6 treatments	61 (31 M, 30 F) Healthy BMI	2-weeks	Breakfasts including: (i) 30 g/d peeled hazelnut paste (ii) 30 g/d unpeeled hazelnut paste (iii) snack with 30 g/d peeled hazelnut paste (iv) snack with 2.5 g/d cocoa powder (v) Snack with 30 g/d peeled hazelnut paste and 2.5 g/d cocoa powder (vi) no snack control group	Peak systolic velocities (PSV), using Doppler ultrasound, at rest vs. control (i) Change: +80.5% ^a^ (ii) Change: +16.9% (iii) Change: +33.7% (iv) Change: +31.5% (v) Change: +26.4% Compared to the control group PSV at rest increased significantly in the peeled hazelnut paste group (57.8%, *p* = 0.04); the unpeeled hazelnut group (56.9%, *p* = 0.04); the snack with peeled hazelnut paste group (95.1%, *p* = 0.002); the peeled hazelnuts and cocoa powder group (68.5%, *p* = 0.01). No significant differences between the snack group with 2.5 g/d cocoa powder and control Peak systolic velocities (PSV) after 3 min of occlusion: (i) Change: +102.7% (ii) Change: +15.6% (iii) Change: +60.7% (iv) Change: −7.1% (v) Change: +64.7% Compared to the control, there were significant increases in the snack with 30 g/d of peeled hazelnut (67.3%, *p* = 0.002); and in the snack with 30 g/d peeled hazelnut paste and 2.5 g cocoa powder group (22.9%, *p* = 0.04). Erythrocyte sedimentation rate and hs-CRP No between-group differences for ESR or hs-CRP Heart rate No significant differences in heart rate N.B. Data was only presented for treatments vs. control, i.e., no other between-group comparisons were reported. Actual follow-up values NR. Actual end of study values for Erythrocyte sedimentation rate, hs-CRP, and heart rate NR
Damavandi et al., 2012 [37]	Randomised parallel 2 treatments	50 ((16 M, 34 F) with type 2 diabetes	8 weeks	(i) Control: no hazelnuts (ii) 10% of total energy hazelnuts	Total antioxidant capacity using colorimetric methods (i) Baseline: 11.19 U/mL End: 9.47 U/mL Change: −1.72 U/mL ^c^ (ii) Baseline: 11.39 U/mL End: 974 U/mL Change: −1.65 U/mL ^b^ No significant differences between-groups hs-CRP (i) Baseline: 1.14 mg/L End: 1.68 mg/L Change: +0.54 mg/L (ii) Baseline: 1.39 mg/L End: 1.17 mg/L Change: −0.22 mg/L No significant differences between-groups Paraoxonase−1 activity (i) Baseline: 68.01 U/mL End: 70.47 U/mL Change: +2.47 U/mL (ii) Baseline: 66.38 U/mL End: 64.55 U/mL Change: −1.73 U/mL No significant differences between-groups
Di Renzo et al., 2014 [52]	Sequential intervention periods 2 treatments	24 BMI ≥ 19 kg/m^2^	4 weeks	(i) 4-week standard diet (Italian Mediterranean diet) (ii) 4-week standard diet with hazelnuts 40 g/d	Oxidised LDL using ELISA kits (i) study end: 40.38 U/L (ii) study end: 36.99 U/L ^b^ Oxidised LDL was significantly lower after hazelnut diet compared to after standard diet (*p* < 0.05). Gene expression was assessed using Quantitative Real-Time PCR (RT2 Profiler PCR assays The following genes were upregulated after hazelnut consumption ^a^: BNIP3, GPX2, GSR, HSPAIA, TTN, TXNRDI The following genes were downregulated after hazelnut consumption ^a^: CCL5, KRTI, MBL2, PRDX6, SODI
Di Renzo et al., 2019 [53]	Single intervention Pilot	24 (14 M, 10 F) healthy	6 weeks	(i) Hazelnuts 40 g/d	Gene expression was assessed using Quantitative Real Time PCR (RT2 Profiler PCR assays There was significant upregulation in the following genes after consuming hazelnuts ^a^: superoxide dismutase (SODI) and catalase (CAT), macrophage migration inhibitory factor (MFI), peroxisome proliferator-activated receptor gamma (PPARγ), vitamin D receptor (VDR), methylenetetrahydrofolate reductase (MTHFR), angiotensin I-converting enzyme (ACE)—all involved in antioxidant and/or anti-inflammatory pathways No significant change in the expression of the following genes after consuming hazelnuts: apolipoprotein E (APOE), interleukin 6 receptor (IL6R), nuclear factor of kappa light polypeptide gene enhancer in B-cell 1 (NFKB1), insulin-like growth factor 2 receptor (IFG2R), upstream transcription factor 1 (USF1)
Durak et al., 1999 [54]	Single intervention	30 (18 M, 12 F) Healthy Medical students	1 month	(i) Hazelnuts (1 g/kg BW (68–69 g))	Antioxidant potential by measuring TBARS (1/nmol/mL·h): (i) Baseline: 0.09, Hazelnut: 0.11, Δ: +0.02 ^c^ Plasma malondialdehyde quantified as tissue thiobarbituric acid-reactive material (nmol/mL): (i) Baseline: 1.33, Hazelnuts: 0.99, Δ: −0.34 ^c^
Guaraldi et al., 2018 [42]	Parallel intervention	60 children and adolescents (mean age 11.6 ± 2.6 years) with hyperlipidaemia	8 weeks	(i) Control (No nuts) (ii) Hazelnuts with skin (15–30 g/d) (iii) Hazelnuts without skin (15–30 g/d)	DNA strand breaks using COMET assay Using endonuclease buffer (%DNA in tail) (i) Baseline: 17.44% End: 13.65% Change: −3.65% ^a^ (ii) Baseline: 18.66% End: 13.41% Change: −5.25% ^a^ (iii) Baseline: 19.70% End: 16.00% Change: −3.70% ^a^ No differences between treatments. DNA strand breaks using phosphate buffer saline (%DNA in tail) (i) Baseline: 6.85% End: 6.25% Change: −0.60% (ii) Baseline: 6.53% End: 6.83% Change: +0.30% (iii) Baseline: 6.15% End: 6.64% Change: +0.49% No differences between treatments. FPG-sensitive sites in PBMCs measured using the enzyme formamidopyrimidine DNA glycosylase (% DNA in tail) (i) Baseline: 15.9% End: 18.9% Change: +3.0% ^a^ (ii) Baseline: 14.7% End: 10.5% Change: −4.2% ^b^ (iii) Baseline: 13.9% End: 10.1% Change: −3.8% ^b^ Significant between-group differences (*p* = 0.001) between the 2 hazelnut groups and the control group. H_2_O_2_-induced DNA damage using COMET assay (% DNA in tail) (i) Baseline: 35.3% End: 29.6% Change: −5.7% (ii) Baseline: 36.6% End: 28.7% Change: −7.9% ^b^ (iii) Baseline: 37.4% End: 32.0% Change: −5.4% No significant differences between treatments Oxidised LDL by ELISA (i) Baseline: 54.1 U/L End: 55.1 U/L Change: 1.0 U/L (ii) Baseline: 54.5 U/L End: 53.3 U/L Change: −1.2 U/L (iii) Baseline: Not measured End: Not measured Change: Not measured No significant differences between treatments
Mercanligil et al., 2007 [55]	Sequential intervention periods 2 treatments	15 (15 M, 0 F) Hyper-cholesterolaemic	4 weeks	(i) Control LF, low cholesterol, high CHO diet	Vascular endothelium function by Doppler ultrasound (i) Baseline: NR, Control: NR, Change: NR (ii) Baseline: NR, Hazelnuts: NR, Change: NR
				(ii) Control + Hazelnuts (40 g/d)	There were no significant differences in endothelial function between the groups.
Michels et al., 2018 [56]	Single intervention	32 (10 M, 22F F) healthy, non-frequent nut consumers, Vit E intake <10 mg a-tocopherol/d, no Vit E supplements in previous 12 months	16 weeks	Baseline (i) Hazelnuts, dry roasted (~57 g/d)	No significant change in serum hs-CRP No significant change in plasma α-tocopherol or ɣ-tocopherol, mmol/mol lipid (Mol lipid = total cholesterol + TGs), measured using HPLC Urinary α-carboxyethyl hydroxychomanol and g-carboxyethyl hydroxychomanol (used to assess Vit E), measured using mass spectroscopy: α-CECH Baseline: 0.844 mmol/g creatinine, Hazelnut diet: 1.14 mmol/g creatinine Δ = +0.296 ^c^ No significant change in ɣ-CECHe from baseline Lymphocyte proliferation assay micronutrient profile, percentage of control cells (data are presented as the proliferation rates of cells in test media compared to control (complete) media): No significant change in α-tocopherol or ɣ-tocopherol (µM) from baseline. Total antioxidant function: Baseline: 56, Hazelnut diet: 60, Change = +4 ^a^
Orem et al., 2013 [57]	Double control sandwich model intervention	21 (18 M, 3 F) Hyper-cholesterolaemic	4-weeks	(i) 4 week no-nut (Control I) diet (ii) 4 week hazelnut-enriched diet (49–86 g/d (18–20% TER)) (iii) 4 week no-nut (Control II) diet	Flow mediated dilation (%) measured using vascular ultrasound of the brachial artery: (i) Control I diet: 15.2% (ii) Hazelnut: 21.8%, Change: +56.6% (iii) Control II diet: 15.9%, Change: −24.6% There was a significant difference in flow-mediated dilation between (i) and (ii), and between (ii) and (iii). There was no significant difference between (i) and (iii). Hs- CRP (mg/L) ^‡^ measured by immunophelometric method: (i) Control I diet: 1.30 mg/L (ii) Hazelnut: 0.7 mg/L, Change: −35.9 (iii) Control II diet: 0.90 mg/L, Change: +71.1% There was a significant difference in Hs-CRP between (i) and (ii), and between (ii) and (iii). There was no significant difference between (i) and (iii). Oxidised-LDL (U/L) measured using commercial ELISA kits: (i) Control I diet: 106 U/L (ii) Hazelnut: 93 U/L, Change: −9.25% (iii) Control II diet: 102 U/L, Change: +9.77.6% There was a significant difference in oxidized-LDL between (i) and (ii), and between (ii) and (iii). There was no significant difference between (i) and (iii). sICAM-1 (ng/mL) measured using commercial ELISA kits: (i) Control I diet: 236 ng/mL (ii) Hazelnut: 216 ng/mL, Change: −8.08% (iii) Control II diet: 234 ng/mL, Change: 6.8% There was a significant difference in sICAM-1 between (i) and (ii), and between (ii) and (iii). There was no significant difference between (i) and (iii). sVCAM-1 (ng/mL) measured using commercial ELISA kits: (i) Control I diet: 981 ng/mL (ii) Hazelnut: 864 ng/mL, Change: −10.6% (iii) Control II diet: 1025 ng/mL, Change: +18.4% There was a significant difference in sVCAM-1 between (i) and (ii), and between (ii) and (iii). There was no significant difference between (i) and (iii). Adiponectin measured using commercial ELISA kits: (i) Control I diet: 4598 ng/mL (ii) Hazelnut: 5615 ng/mL, Change: +29.1% (iii) Control II diet: 5057 ng/mL, Change: −5.15% There was a significant difference in adiponectin between (i) and (ii). There was no significant difference between any other treatments. Plasma α-tocopherol (mg/L) determined by HPLC: (i) Control I diet: 11.7 mg/L (ii) Hazelnut: 13.7 mg/L, Change: 16.9% (iii) Control II diet: 13.1 mg/L, Change: −2.24% There was a significant difference in plasma α-tocopherol between (i) and (ii), and between (i) and (iii). There was no significant difference between (ii) and (iii). α-tocopherol in LDL, determined by HPLC (µg/mg LDL protein): (i) Control I diet: 4.71 µg/mg (ii) Hazelnut: 5.76 µg/mg, Change: 24.5% (iii) Control II diet: 4.41 µg/mg, Change: −22.3% There was a significant difference in α-tocopherol in LDL between (i) and (ii) and between (ii) and (iii). There was no significant difference between (i) and (iii). Vitamin B12 (pg/mL) measured by enzymatic methods: (i) Control I diet: 375 pg/mL (ii) Hazelnut: 386 pg/mL, Change: +2.94% (iii) Control II diet: 334 pg/mL, Change: −13.8% There was a significant difference in vitamin B12 between (i) and (iii), and between (ii) and (iii). There was no significant difference between (i) and (ii). Folic acid (ng/mL), measured by enzymatic methods: (i) Control I diet: 8.58 ng/mL (ii) Hazelnut: 9.08 ng/mL, Change: +6.24% (iii) Control II diet: 8.04 ng/mL, Change: −11.3% There was a significant difference in folic acid between (ii) and (iii). There was no significant difference between the other treatments. There was no significant difference in endothelin-1 (fmol/mL) or homocysteine (µmol/L) across any of the treatments, overall *p*-value *p* = 0.651 and *p* = 0.484 respectively. *N.B. p-values for between-group differences NR.*
Santi et al., 2017 [58]	Double control sandwich model intervention	24 (14 M, 10 F) Healthy BMI > 19 kg/m^2^, <30 kg/m^2^	6-weeks	(i) 2-week ‘standard’ diet (ii) 6-week raw hazelnut (40 g/d) (iii) 6-week ‘standard’ diet ‘washout’	Uric acid (mg/dL) measured by uricase and peroxidase reactions: (i): 4.66 (ii): 4.31, Change: −0.35 (iii): 4.66, Change: +0.35 There was a significant (i) vs. (ii) *p =* 0.025 (i) vs. (iii) *p =* 0.99 (ii) vs. (iii) *p =* 0.013 Serum creatinine (mg/dL) measured by creatinine amidohydrolase, sarcosine oxidase and peroxidase reactions: (i): 0.94 (ii): 0.93, Change: −0.01 (iii): 0.82, Change: −0.11 (i) vs. (ii) *p =* 0.29 (i) vs. (iii) *p ≤* 0.001 (ii) vs. (iii) *p =* 0.001 Alanine aminotransferase (ALT) (U/L) measured by latticodehydrogenase reactions: (i): 30.09 (ii): 35.22, Change: +5.13 (iii): 31.52, Change: −3.70 (i) vs. (ii) *p =* 0.011 (i) vs. (iii) *p =* 0.065 (ii) vs. (iii) *p =* 0.99 Gamma-glutamyl transferase (GGT) (U/L) measured by oxaloacetate decarboxylase, pyruvate oxidase and peroxidase reactions: (i): 38.04 (ii): 35.27, Change: −2.77 (iii): 36.26, Change: +0.99 (i) vs. (ii) *p =* 0.001 (i) vs. (iii) *p =* 0.31 (ii) vs. (iii) *p =* 0.16 There was no significant difference in AST, serum iron, azotaemia, total bilirubin, Hb, WBCs, RBC, platelet count, or total plasma protein content between any of the treatment groups.
Tey et al., 2011 [43]	Randomised Crossover 3 treatments	48 (20 M, 28 F) Mildly hyper-cholesterolaemic	4 weeks	(i) Ground hazelnuts (30 g/d) (ii) Sliced hazelnuts (30 g/d) (iii) Whole hazelnuts (30 g/d)	α-tocopherol measured using HPLC (mmol/L): (i) Baseline: 33.1, Ground: 34.7, Change: +1.6 ^b^ (ii) Baseline: 33.1, Sliced: 34.2, Change: +1.1 ^b^ (iii) Baseline: 33.1, Whole: 34.2, Change: +1.1 ^b^ There was no significant difference in α-tocopherol between different forms of nuts.
Tey et al., 2013 [47]	Randomised Parallel 3 treatments	107 (46 M, 61 F) Overweight and obese individuals with a BMI ≥ 25 kg/m^2^	12 weeks	(i) Control group (no hazelnuts) (ii) Hazelnuts (30 g/d) (iii) Hazelnuts (60 g/d)	α-tocopherol measured using HPLC (µmol/L): (i) Baseline: 24.3, Control: 24.2, Change: −0.01 (ii) Baseline: 25.4, 30 g/d: 24.6, Change: −0.08 (iii) Baseline: 24.5, 60 g/d: 25.1, Change: +0.6 There was no significant difference in α-tocopherol between the treatments. Hs-CRP^ measured using a CRP Unimate kit (mg/L): (i) Baseline: 1.93, Control: 1.75, Change: N/R (ii) Baseline: 1.47, 30 g/d: 1.45, Change: N/R (iii) Baseline: 1.51, 60 g/d: 1.37, Change: N/R There was no significant difference in Hs-CRP between the treatments. IL-6^ measured using ELISA kits (pg/mL): (i) Baseline: 1.37, Control: 1.52, Change: N/R (ii) Baseline: 1.28, 30 g/d: 1.30, Change: N/R (iii) Baseline: 1.74, 60 g/d: 1.49, Change: N/R There was no significant difference in IL-6 between the treatments. ICAM-1 measured using ELISA kits (µg/L): (i) Baseline: 208, Control: 204, Change: −4 (ii) Baseline: 221, 30 g/d: 206, Change: −15 ^a^ (iii) Baseline: 207, 60 g/d: 195, Change: +12 ^a^ There was no significant difference in ICAM-1 between the treatments. VCAM-1 measured using ELISA kits (µg/L): (i) Baseline: 571, Control: 567, Change: −4 (ii) Baseline: 652, 30 g/d: 644, Change: −8 (iii) Baseline: 628, 60 g/d: 586, Change: −42 There was a tendency toward improvement in VCAM-1 in the 60 g/d hazelnut group (*p* = 0.07).
Tey et al., 2015 [59]	Single intervention	20 Māori (8 M, 12 F) and 19 (5 M, 14 F) European aged above 18 years	4 weeks	(i) Raw hazelnuts (30 g/d)	Hs-CRP ^ measured using a CRP Unimate kit (mg/L): Māori: (i) Baseline: 0.42, Hazelnuts: 0.70, Change: N/R Europeans: (i) Baseline: 0.69, Hazelnuts: 0.83, Change: N/R
Tey et al., 2017 [49]	Randomised Crossover 2 treatments	72 (24 M, 48 F) Aged 18 years and above	4 weeks	(i) Raw hazelnuts (30 g/d) (ii) Dry roasted, lightly salted hazelnuts (30 g/d)	α-tocopherol measured using HPLC (µmol/L): (i) Baseline: 30.2, Raw: 31.42, Change: +1.22 ^b^ (ii) Baseline: 30.2, Lightly salted: 31.26, Change: +1.06 There was no significant difference in α-tocopherol between the treatments.
Yucesan et al., 2010 [60]	Single intervention	21 (8 M, 13 F) Normolipidaemic	4 weeks	(i) Hazelnuts (1 g/kg BW (49–86 g))	α-tocopherol in LDL (µg/mg LDL protein), measured using HPLC: (i) Baseline: 4.82, Hazelnuts: 5.35, Change: +0.53 ^a^ Oxidised LDL (U/L): (i) Baseline: 57.2, Hazelnut: 48.2, Change: −9.0 ^b^ Hs-CRP (mg/dL), measured using immunophrelometric method: (i) Baseline: 0.13, Hazelnut: 0.11, Change: −0.02 sVCAM-1 (ng/mL), measured using ELISA kits: (i) Baseline: 478, Hazelnut: 446, Change: −32 Endothelin-1 (fmol/mL), measured using ELISA kits: (i) Baseline: 2.04, Hazelnut: 1.99, Change: −0.05

Abbreviations used: BW, body weight; CHEC, carboxyethyl hydrochromanol; CHO, carbohydrate; ELISA, enzyme-linked immunosorbent assay; F, female; HPLC, high-performance liquid chromatography; hs-CRP, high-sensitivity C reactive protein; ICAM-1, intracellular adhesion molecule-1; LDL, low-density lipoprotein; LF, low fat; M, male; NR, not reported; TER, total energy requirement; sVCAM-1, soluble vascular adhesion molecule-1. All values are arithmetic means unless otherwise stated. ^1^ Change (within-group) = Post-treatment value minus Pre-treatment value (i.e., baseline); ^a^ *p* < 0.05; ^b^ *p* < 0.01; ^c^ *p* < 0.001; only for those which reported within-group change. ^ Geometric mean. ^‡^ Median.

**Table 10:** Dietary intervention trials investigating the effects of nut consumption on acceptance (*n* = 7).

Author, Year	Study Design	Subjects	Measurement; Timepoint	Treatments; Number of Exposures	Results ^1^
Devi et al., 2016 [40]	Randomised Crossover 4 treatments	32 (11 M 21 F) healthy	Desire to consume on a 150 mm VAS; Measured daily during the exposure period	(i) Bread containing 30 g finely sliced hazelnuts per 120 g; Exp. period = 5 d (ii) Bread containing 30 g defatted hazelnut flour per 120 g; Exp. period = 5 d (iii) Bread containing 15 g finely sliced defatted hazelnuts and 15 g hazelnut flour per 120 g; Exp. period = 5 d (iv) Control white bread with no nuts; Exp. period = 5 d	*5-day exposure period* (i) Stable: ^a^ (ii) Stable: ^c^ (iii) Stable: ^a^ (iv) Stable: ^b^
			Overall liking on a 150 mm VAS; Measured daily during the exposure period	(i) Bread containing 30 g finely sliced hazelnuts per 120 g; Exp. period = 5 d (ii) Bread containing 30 g defatted hazelnut flour per 120 g; Exp. period = 5 d (iii) Bread containing 15 g of finely sliced defatted hazelnuts and 15 g hazelnut flour per 120 g; Exp. period = 5 d (iv) Control white bread with no nuts; Exp. period = 5 d	*5-day exposure period* (i) Stable: ^a^ (ii) Stable: ^c^ (iii) Stable: ^b^ (iv) Stable: ^b^ *Pre*- vs. *Post*- (i) No significant change: 74.8 ^b^ vs. 79.3 ^b^ (ii) No significant change: 46.5 ^a^ vs. 41.4 ^a^ (iii) Significant increase: 53.4 ^a^ vs. 66.4 ^c^ (*p* < 0.05) (iv) No significant change: 44.5 ^a^ vs. 46.5 ^a^
Tey et al., 2011 [44]	Randomised Crossover 3 treatments	20 M, 28 F	Desire to consume on a 150 mm VAS; Measured daily during the exposure period	(i) Ground hazelnuts (30 g/d); Exp. period = 28 d (ii) Sliced hazelnuts (30 g/d); Exp. period = 28 d (iii) Whole hazelnuts (30 g/d); Exp. period = 28 d	*28-day exposure period* (i) Stable: 92.1 ^a^ (ii) Stable: 107.7 ^b^ (iii) Stable: 116.2 ^b^
			Overall liking on a 150 mm VAS; Measured daily during the exposure period and at pre- and post-exposure	(i) Ground hazelnuts (30 g/d); Exp. period = 28 d (ii) Sliced hazelnuts (30 g/d); Exp. period = 28 d (iii) Whole hazelnuts (30 g/d); Exp. period = 28 d	*28-day exposure period* (i) Stable: 100.8 ^a^ (ii) Stable: 109.9 ^b^ (iii) Stable: 117.7 ^b^ *Pre*- vs. *Post*- (i) No significant change: 92.8 ^a^ vs. 87.4 ^a^ (ii) No significant change: 109.1 ^b^ vs. 107.3 ^b^ (iii) No significant change: 113.7 ^b^ vs. 110.2 ^b^
Tey et al., 2012 [46]	Randomised Parallel 4 treatments	55 M, 63 F	Desire to consume on a 100 mm VAS; Measured daily during the exposure period	(i) Hazelnuts (42 g/d); Exp. period = 84 d (ii) Chocolate (50 g/d); Exp. period = 84 d (iii) Potato crisps (50 g/d); Exp. period = 84 d	*84-day exposure period* (i) Stable: 60.9 ^a^ (ii) Stable: 64.9 ^a^ (iii) Stable: 62.7 ^a^
			Overall liking on a 100 mm VAS; Measured daily during the exposure period and at pre- and post-exposure	(i) Hazelnuts (42 g/d); Exp. period = 84 d (ii) Chocolate (50 g/d); Exp. period = 84 d (iii) Potato crisps (50 g/d); Exp. period = 84 d	*84-day exposure period* (i) Stable: 57.9 ^a^ (ii) Decrease over time: −9.9 ^a^ (*p* = 0.002) (iii) Decrease over time: −8.6 ^a^ (*p* = 0.031) *Pre*- vs. *Post*- (i) No significant change: 61.1 ^a^ vs. 53.8 ^a^ (ii) Significant decrease: 76.2 ^a^ vs. 53.6 ^a^ (*p* < 0.001) (iii) No significant change: 67.0 ^a^ vs. 58.0 ^a^
Tey et al., 2013 [47]	Randomised Parallel 3 treatments	107 (46 M, 61 F) Overweight and obese individuals with a	Desire to consume on a 150 mm VAS; Measured daily during the exposure period	(i) Hazelnuts (30 g/d); Exp. period = 84 d (ii) Hazelnuts (60 g/d); Exp. period = 84 d	*84-day exposure period* (i) Increase over time: +14.2 ^a^ (*p* = 0.003) (ii) Decrease over time: −29.4 ^b^ (*p* < 0.001)
		BMI ≥ 25 kg/m^2^	Overall liking on a 150 mm VAS; Measured daily during the exposure period and at pre- and post-exposure	(i) Hazelnuts (30 g/d); Exp. period = 84 d (ii) Hazelnuts (60 g/d); Exp. period = 84 d	(i) *84-day exposure period* (i) Stable: +0.4 ^a^ (ii) Decrease over time: −24.4 ^b^ (*p* < 0.001) *Pre*- vs. *Post*- (i) vs. (ii): +14.6 (*p* < 0.05)
Tey et al., 2015 [59]	Single intervention	20 Māori (8 M, 12 F) and 19 (5 M, 14 F) European	Desire to consume on a 150 mm VAS; Measured daily during the exposure period	(i) Māori: Hazelnuts (30 g/d), Exp. period = 28 d (ii) European: Hazelnuts (30 g/d), Exp. period = 28 d	*28-day exposure period* (i) No significant change (ii) No significant change
		aged above 18 years	Overall liking on a 150 mm VAS; Measured daily during the exposure period	(i) Māori: Hazelnuts (30 g/d), Exp. period = 28 d (ii) European: Hazelnuts (30 g/d), Exp. period = 28 d	*28-day exposure period* (i) No significant change (ii) No significant change *Pre*- vs. *Post*- (i) vs. (ii): No difference
Tey et al., 2015 [48]	Randomised Crossover 6 treatments (only 3 hazelnut treatments reported)	74 (34 M, 40 F) healthy participants	Desire to consume on a 150 mm VAS; Measured daily during the exposure period	(i) Ground hazelnuts (30 g/d); Exp. period = 5 d (ii) Sliced hazelnuts (30 g/d); Exp. period = 5 d (iii) Whole hazelnuts (30 g/d); Exp. period = 5 d	*5-day exposure period* (i) Stable: ^a^ (ii) Stable: ^b^ (iii) Stable: ^c^
			Overall liking on a 150 mm VAS; Measured daily during the exposure period and at pre- and post-exposure	(i) Ground hazelnuts (30 g/d); Exp. period = 5 d (ii) Sliced hazelnuts (30 g/d); Exp. period = 5 d (iii) Whole hazelnuts (30 g/d); Exp. period = 5 d	*5-day exposure period* (i) Stable: ^a^ (ii) Stable: ^b^ (iii) Stable: ^c^
Tey et al., 2017 [49]	Randomised Crossover 2 treatments	72 (24 M, 48 F) Aged 18 years and above	Desire to consume on a 150 mm VAS; Measured daily during the exposure period	(i) Raw hazelnuts (30 g/d); Exp. period = 28 d (ii) Dry roasted, lightly salted hazelnuts (30 g/d); Exp. period = 28 d	*28-day exposure period* (i) Stable: ^a^ (ii) Stable: ^a^
			Overall liking on a 150 mm VAS; Measured daily during the exposure period and at pre- and post-exposure	(i) Raw hazelnuts (30 g/d); Exp. period = 28 d (ii) Dry roasted, lightly salted hazelnuts (30 g/d); Exp. period = 28 d	*28-day exposure period* (i) Stable: ^a^ (ii) Stable: ^a^ *Pre*- vs. *Post*- (i) No significant change: 105 ^a^ vs. 108 ^a^ (ii) No significant change: 107 ^a^ vs. 111 ^a^

Abbreviations used: Exp., exposure; F, female; M, male; No., number; VAS, visual analogue scale. All values are arithmetic means unless otherwise stated. ^1^ No acceptance results for no nut control group. Results: ^a, b, c^ Between-group comparisons, determined using ANOVA or regression models (*p* < 0.05).

## Data Availability

Not applicable.

## Supplementary Materials

The following supporting information can be downloaded at: https://www.mdpi.com/article/10.3390/ijerph19052880/s1, Table S1: Search terms.
