# Supplementary material for: Effects of Hazelnut Consumption on Cardiometabolic Risk Factors and Acceptance: A Systematic Review
Source: Int J Environ Res Public Health. 2022 Mar 1;19(5):2880. doi: 10.3390/ijerph19052880 (PMC8910742; doi:10.3390/ijerph19052880)
Supplement: Supplementary file 1 [file ijerph-19-02880-s001.zip › ijerph-1511173-supplementary.pdf]

Table S1. Search terms

Order	Terms
#1	“hazelnut*” or “filbert*” or “cobnut*” or “corylus avellana”
#2	"lipid*" or "cholesterol" or “lipoprotein*” or "apolipoprotein*" or “apo” or “HDL” or “LDL” or “tri-glyc*”
#3	"heart disease" or "cardiovascular disease" or “coronary heart disease” or “ischaemic heart disease” or “ischemic heart disease” or “CVD” or “CHD” or “IHD”
#4	"glycaemic control" or "glycemic control” or "glucose" or "insulin" or "HbA1c" or “glycated haemoglo-bin” or “glycated hemoglobin” or "HOMA-IR" or “homeostatic model assessment” or “homeostasis model assessment” or “diabet*” or “metabolic syndrome”
#5	"hypertens*" or "stroke" or “blood pressure” or “systolic” or “diastolic”
#6	"tocopherol*" or “tocotrienol*“ or “vitamin E”
#7	"antioxid*" or "oxidati*" or "inflammat*" or "endotheli*" or “CRP” or “C reactive protein” or “fibrino-gen” or “white blood cell*” or “neutrophil*” or “interleukin*” or “cytokine*” or “ICAM” or “VCAM” or “adhesion molecule” or ”I-CAM” or “V-CAM” or “IL-6” or “IL-1” or “IL-10”
#8	“body weight” or “body mass index” or “BMI” or “obes*” or “overweight” or “waist” or “adipos*” or “body fat”
#9	“acceptance” or “liking” or “desire to consume” or “desire to eat” or “sati*” or “appetite”
